# Pediatric Gastrointestinal Tract Outcomes During the Postacute Phase of COVID-19

**DOI:** 10.1001/jamanetworkopen.2024.58366

**Published:** 2025-02-07

**Authors:** Dazheng Zhang, Ronen Stein, Yiwen Lu, Ting Zhou, Yuqing Lei, Lu Li, Jiajie Chen, Jonathan Arnold, Michael J. Becich, Elizabeth A. Chrischilles, Cynthia H. Chuang, Dimitri A. Christakis, Daniel Fort, Carol R. Geary, Mady Hornig, Rainu Kaushal, David M. Liebovitz, Abu S. M. Mosa, Hiroki Morizono, Parsa Mirhaji, Jennifer L. Dotson, Claudia Pulgarin, Marion R. Sills, Srinivasan Suresh, David A. Williams, Robert N. Baldassano, Christopher B. Forrest, Yong Chen

**Affiliations:** 1 The Center for Health AI and Synthesis of Evidence, University of Pennsylvania, Philadelphia; 2 Department of Biostatistics, Epidemiology and Informatics, Perelman School of Medicine at the University of Pennsylvania, Philadelphia; 3 Division of Gastroenterology, Hepatology, and Nutrition, Children’s Hospital of Philadelphia, Philadelphia, Pennsylvania; 4 Department of Pediatrics, Perelman School of Medicine at the University of Pennsylvania, Philadelphia; 5 Applied Mathematics and Computational Science, School of Arts and Sciences, University of Pennsylvania, Philadelphia; 6 Division of General Internal Medicine, University of Pittsburgh School of Medicine, Pittsburgh, Pennsylvania; 7 Department of Biomedical Informatics, University of Pittsburgh School of Medicine, Pittsburgh, Pennsylvania; 8 Department of Epidemiology, College of Public Health, University of Iowa, Iowa City; 9 Division of General Internal Medicine, Penn State College of Medicine, Hershey, Pennsylvania; 10 Center for Child Health, Behavior, and Development, Seattle Children’s Research Institute, Seattle, Washington; 11 Ochsner Center for Outcomes Research, Ochsner Health, New Orleans, Louisiana; 12 College of Medicine, University of Nebraska Medical Center, Omaha; 13 Department of Epidemiology, Columbia University Mailman School of Public Health, New York, New York; 14 Department of Population Health Sciences, Weill Cornell Medical College, New York, New York; 15 Division of General Internal Medicine, Feinberg School of Medicine, Northwestern University, Chicago, Illinois; 16 Department of Biomedical Informatics, Biostatistics and Medical Epidemiology, University of Missouri School of Medicine, Columbia; 17 Center for Genetic Medicine Research, Children’s National Hospital, Washington, DC; 18 Institute for Clinical Translational Research, Albert Einstein College of Medicine, New York, New York; 19 Division of Pediatric Gastroenterology, Hepatology, and Nutrition, Arkansas Children’s Hospital, University of Arkansas for Medical Sciences, Little Rock; 20 Department of Population Health, New York University Grossman School of Medicine, New York, New York; 21 Department of Research, OCHIN, Inc, Portland, Oregon; 22 Department of Pediatrics, University of Colorado School of Medicine and Children’s Hospital Colorado, Aurora; 23 Division of Health Informatics, Department of Pediatrics, University of Pittsburgh School of Medicine, Pittsburgh, Pennsylvania; 24 Division of Emergency Medicine, Department of Pediatrics, University of Pittsburgh School of Medicine, Pittsburgh, Pennsylvania; 25 UPMC Children’s Hospital of Pittsburgh, Pittsburgh, Pennsylvania; 26 Department of Anesthesiology, University of Michigan, Ann Arbor; 27 Applied Clinical Research Center, Children’s Hospital of Philadelphia, Philadelphia, Pennsylvania; 28 Penn Institute for Biomedical Informatics, Philadelphia, Pennsylvania; 29 Leonard Davis Institute of Health Economics, Philadelphia, Pennsylvania; 30 Penn Medicine Center for Evidence-based Practice, Philadelphia, Pennsylvania

## Abstract

**Importance:**

The profile of gastrointestinal (GI) tract outcomes associated with the postacute and chronic phases of COVID-19 in children and adolescents remains unclear.

**Objective:**

To investigate the risks of GI tract symptoms and disorders during the postacute (28-179 days after documented SARS-CoV-2 infection) and the chronic (180-729 days after documented SARS-CoV-2 infection) phases of COVID-19 in the pediatric population.

**Design, Setting, and Participants:**

This retrospective cohort study was performed from March 1, 2020, to September 1, 2023, at 29 US health care institutions. Participants included pediatric patients 18 years or younger with at least 6 months of follow-up. Data analysis was conducted from November 1, 2023, to February 29, 2024.

**Exposures:**

Presence or absence of documented SARS-CoV-2 infection. Documented SARS-CoV-2 infection included positive results of polymerase chain reaction analysis, serological tests, or antigen tests for SARS-CoV-2 or diagnosis codes for COVID-19 and postacute sequelae of SARS-CoV-2.

**Main Outcomes and Measures:**

GI tract symptoms and disorders were identified by diagnostic codes in the postacute and chronic phases following documented SARS-CoV-2 infection. The adjusted risk ratios (ARRs) and 95% CI were determined using a stratified Poisson regression model, with strata computed based on the propensity score.

**Results:**

The cohort consisted of 1 576 933 pediatric patients (mean [SD] age, 7.3 [5.7] years; 820 315 [52.0%] male). Of these, 413 455 patients had documented SARS-CoV-2 infection and 1 163 478 did not; 157 800 (13.6%) of those without documented SARS-CoV-2 infection had a complex chronic condition per the Pediatric Medical Complexity Algorithm. Patients with a documented SARS-CoV-2 infection had an increased risk of developing at least 1 GI tract symptom or disorder in both the postacute (8.64% vs 6.85%; ARR, 1.25; 95% CI, 1.24-1.27) and chronic (12.60% vs 9.47%; ARR, 1.28; 95% CI, 1.26-1.30) phases compared with patients without a documented infection. Specifically, the risk of abdominal pain was higher in COVID-19–positive patients during the postacute (2.54% vs 2.06%; ARR, 1.14; 95% CI, 1.11-1.17) and chronic (4.57% vs 3.40%; ARR, 1.24; 95% CI, 1.22-1.27) phases.

**Conclusions and Relevance:**

In this cohort study, the increased risk of GI tract symptoms and disorders was associated with the documented SARS-CoV-2 infection in children or adolescents during the postacute or chronic phase. Clinicians should note that lingering GI tract symptoms may be more common in children after documented SARS-CoV-2 infection than in those without documented infection.

## Key Points

**Question:**

Is COVID-19 associated with the risk of gastrointestinal (GI) tract symptoms and diseases during the postacute phase in children and adolescents?

**Findings:**

In this cohort study of 1 576 933 pediatric patients, documented SARS-CoV-2 infection was associated with GI tract symptoms and diseases during the postacute phase. Children and adolescents with documented SARS-CoV-2 infection faced a 25% higher risk of newly diagnosed GI tract symptoms and disorders during the postacute phase compared with those without documented SARS-CoV-2 infection (incidence, 8.64% vs 6.85%).

**Meaning:**

These results suggest that clinicians should note that lingering GI tract symptoms after documented SARS-CoV-2 infection in children may be more common than in those without documented SARS-CoV-2 infection.

## Teaser

This cohort study investigates the risk of gastrointestinal tract symptoms and disorders during the postacute and chronic phases of COVID-19 illness among children and adolescents.

## Introduction

Post–COVID-19 condition, also known as *long COVID* or the postacute sequelae of SARS-CoV-2 (PASC), was initially identified primarily in adults,^1,2,3,4,5,6,7,8,9,10,11^ but its emergence has also raised concerns for the pediatric population.^12,13^ In the US, depending on the definition used and the study population, PASC is reported to affect 1.3% of children, a rate lower than that reported in some studies for adults (6.7%).^14^ This may not be surprising, as there are important differences in the presentation and outcomes of acute SARS-CoV-2 infection between children and adults. Children can have different symptoms compared with adults and tend to have a milder disease course, or even one that is asymptomatic, with a lower risk of hospitalization or death, particularly children without preexisting conditions.^15,16,17^ Given these differences in acute infection, as well as the differences in prevalence between children and adults, the characteristics of PASC require further study in children.

Multiple organ systems can be affected in PASC, including the gastrointestinal (GI) tract.^18,19,20,21,22,23^ The risk of GI tract symptoms and disorders, including abdominal pain, diarrhea, constipation, vomiting, bloating, irritable bowel syndrome (IBS), and gastroesophageal reflux disease (GERD), was increased during the first year after SARS-CoV-2 infection among adults.^10^ This phenomenon of chronic GI tract symptoms potentially developing after infection was well described in adults before the SARS-CoV-2 pandemic, with postinfectious IBS associated with acute gastroenteritis^24,25^ and post–viral gastroparesis seen in association with several respiratory and GI tract viruses.^26^ Children are also at risk for developing an array of lingering GI tract symptoms after an acute infection, but in contrast to adults, pediatric patients predominantly present with chronic abdominal pain and constipation.^27,28^ As the data regarding the long-term outcomes of SARS-CoV-2 in the GI tract among children are limited,^29^ it is unclear whether children have the same risks of GI tract conditions during the postacute phase of COVID-19 as has been seen with adults.

In this retrospective cohort study, we aimed to investigate the risk of GI tract symptoms and disorders during the postacute and chronic phases of COVID-19 illness among children and adolescents from 29 children’s hospitals and health institutions in the US. Specifically, we first estimated the incidence of any GI tract symptoms and disorders associated with documented SARS-CoV-2 infection and evaluated whether the documented SARS-CoV-2 infection is associated with an increased risk of GI tract symptoms and disorders. To our knowledge, our investigation has the longest follow-up duration (mean [SD], 1.85 [0.74] years) and is the largest US study assessing the risks of documented SARS-CoV-2 infection for GI tract symptoms and diseases in children and adolescents.

## Methods

### Data Source

This cohort study was approved by the University of Pennsylvania’s Institutional Review Board with a waiver of informed consent for the use of deidentified data. We followed the Strengthening the Reporting of Observational Studies in Epidemiology (STROBE) reporting guideline, and this study is part of the National Institutes of Health Researching COVID to Enhance Recovery (RECOVER) Initiative. The statistical analysis plan is found in eAppendix 1 in Supplement 1. We included 29 health institutions listed in eAppendix 2 in Supplement 1. The data are standardized according to the National Patient-Centered Clinical Research Network common data model.^30^

### Cohort Construction

**Figure 1.**
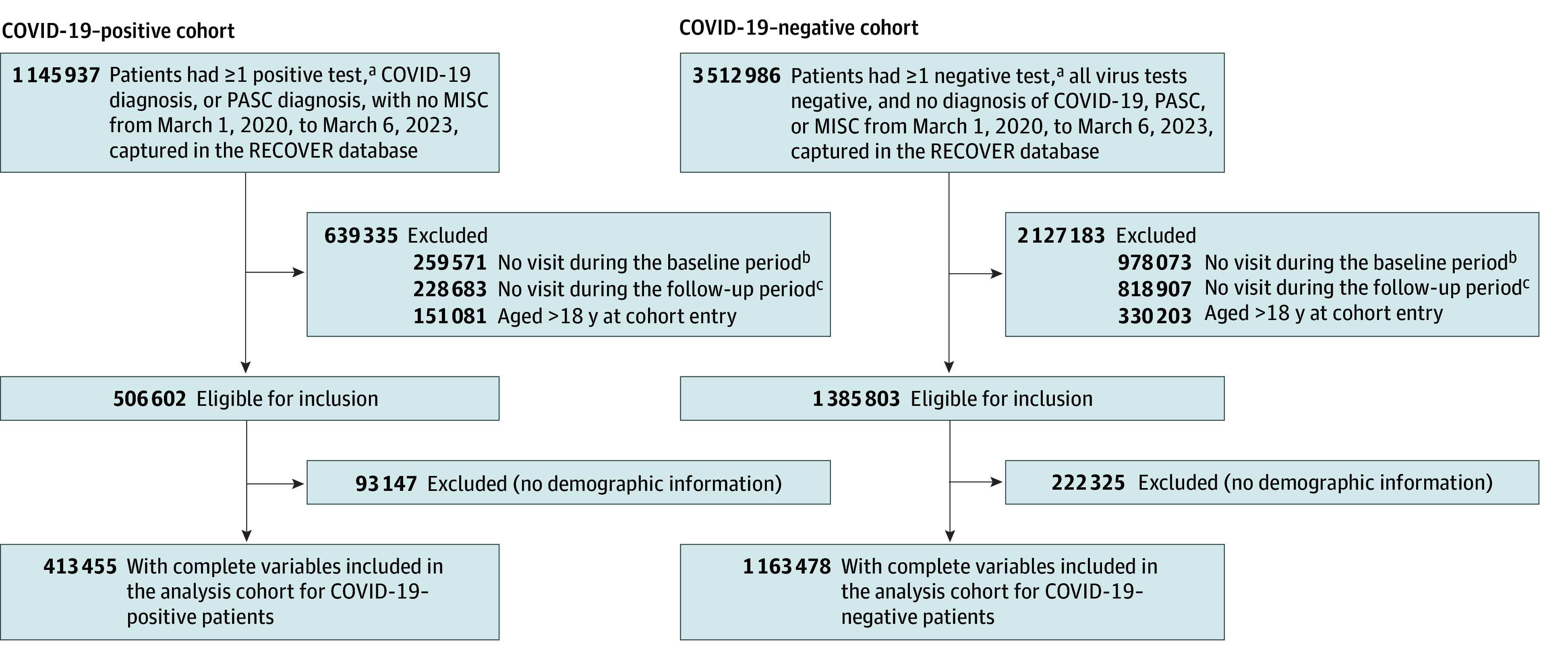
Flowchart of Study Cohort Selection for COVID-19–Positive and COVID-19–Negative Groups Patients were identified with (positive) or without (negative) documented SARS-CoV-2 infection in the Researching COVID to Enhance Recovery (RECOVER) database. MISC indicates multisystem inflammatory syndrome in children; PASC, postacute sequelae of SARS-CoV-2. ^a^Positive results include polymerase chain reaction, antigen, and serological tests. ^b^Indicates 24 months to 7 days before the index date. ^c^Indicates 28 to 179 days before the index date.

Our study period spanned from March 1, 2020, to September 1, 2023, with a cohort entry date cutoff of March 6, 2023 (≥6-month follow-up period for each individual). The index date is defined as the first date of documented SARS-CoV-2 infection for the COVID-19–positive cohort or a randomly selected negative test result for the COVID-19–negative cohort. Our study focused on individuals 18 years or younger who had at least 1 visit within 24 months to 7 days before the index date and subsequently had another encounter 28 to 729 days after the index date. The detailed inclusion-exclusion criteria are summarized in Figure 1. For the COVID-19–positive cohort, we included patients who had at least 1 documented SARS-CoV-2 infection, including positive polymerase chain reaction test results, serological tests, antigen tests, diagnosed COVID-19, or PASC. As for the COVID-19–negative cohort, we selected pediatric patients who had at least 1 negative test result and did not have the documented SARS-CoV-2 infection. Patients diagnosed with multisystem inflammatory syndrome in children were excluded from both the COVID-19–positive and COVID-19–negative cohorts.

### Defining GI Tract Outcomes

We explored 9 predefined GI tract symptoms and disorders within each follow-up period (28-729 days after the cohort entry date). The GI tract signs or symptoms were abdominal pain, bloating, constipation, diarrhea, nausea, and vomiting. The GI tract disorders were functional dyspepsia, GERD, and IBS. The GI tract symptoms and disorders were categorized using the *International Statistical Classification of Diseases, Tenth Revision, Clinical Modification* (*ICD-10-CM*) codes provided in eTable 1 in Supplement 1.

### Patient Characteristics

The baseline covariates included factors such as age at cohort entry, sex (female or male), electronic health record (EHR)–derived race and ethnicity (including Asian American or Pacific Islander, Hispanic, non-Hispanic Black, non-Hispanic White, multiracial, and other [including no information, refuse to answer, or unknown from EHR concept source values]), and obesity status during the baseline period (from 24 months to 7 days before the cohort entry date). Race and ethnicity were included in the analysis because they were potential confounding variables. We also considered the month of cohort entry from March 1, 2020, to March 6, 2023, and chronic condition status as defined by the Pediatric Medical Complexity Algorithm,^31,32^ which categorizes medical conditions into no chronic condition, noncomplex chronic condition, and complex chronic condition. Additionally, we included the site index; use of health care services indicated by the number of inpatient, outpatient, and emergency department visits; the number of unique medications or prescriptions (categorized as 0, 1, 2, or ≥3); and the count of negative test results during the baseline period (grouped as 0, 1, 2, or ≥3). eFigure 1 in Supplement 1 illustrates the structure and possible associations of baseline characteristics, including potential confounders and pathways.

### Sensitivity Analysis

We performed subgroup analyses based on age, race and ethnicity, obesity status, sex, SARS-CoV-2 virus variants (pre-Delta, Delta, and Omicron), hospitalization status (intensive care unit admission, hospitalization, and no hospitalization), and medical history of diabetes (yes or no) or cardiovascular disorders (yes or no). The *ICD-10-CM* codes for diabetes and cardiovascular diseases were listed in eTables 2 and 3 in Supplement 1. We also conducted subgroup analyses by COVID-19 severity (asymptomatic, mild, moderate, or severe) within the acute phase (0-28 days after the cohort entry date).^33^ For the chronic phase, where follow-up time varied, we calculated GI tract symptom incidence using person-years. We conducted separate sensitivity analyses adjusting for GI tract–related visits during the acute phase and GI tract–related medications (eTable 4 in Supplement 1) before the postacute phase. We performed subgroup analyses based on the type of documented index date (positive test result, COVID-19 diagnosis, or PASC diagnosis). Although we have incorporated extensive confounders, the regression model might still be susceptible to residual bias, including unmeasured confounder bias. To reduce such bias from the estimation of ARR, we used a calibration approach^34,35^ (eAppendix 4 in Supplement 1) involving 36 negative control outcomes, such as foreign body in the ear or insect bite (eTable 5 in Supplement 1).

### Statistical Analysis

To illustrate the incidence of new GI tract symptoms and disorders—those not present prior to the acute infection—we calculated the proportion by dividing the number of patients with GI tract symptoms or disorders during the follow-up period but not at baseline by the total number of populations at risk. We investigated the adjusted risk ratio (ARR) of the prespecified GI tract symptoms and disorders during the postacute phase (28-179 days after the cohort entry date) and the chronic phase (180-729 days after the cohort entry date). We investigated the risk of composites of any GI tract symptoms, GI tract disorders, and visits related to GI tract symptoms or disorder during the postacute or the chronic phase. Before fitting the model, we used a cutoff incidence value of 0.1%^36^ to prevent the implications of model overfitting for rare GI tract outcomes. To ensure the covariate balance between the COVID-19–positive and –negative cohorts, we used propensity-score–based stratification. The propensity score was fitted by a logistic regression model with the COVID-19 positivity as outcome and the patient characteristics as covariates. Confounder assessment used standardized mean differences, with a cutoff value set at less than 0.1.^37^ The ARR estimated risk quantification via a modified Poisson regression model^38^ with 6 strata defined by the propensity score. For analysis during the chronic phase, we included person-time as an offset to adjust for individual follow-up time. We applied the Benjamini-Hochberg procedure^39^ to control the false discovery rate for multiple test issues. All analyses were conducted using R, version 4.1.2 (R Program for Statistical Computing), and codes are available in eAppendix 3 in Supplement 1. Statistical significance was set at 2-tailed *P* < .05. Data analysis was conducted from November 1, 2023, to February 29, 2024.

## Results

We present the descriptive analysis of both the COVID-19–positive and –negative cohorts in Table 1. Our analysis included 1 576 933 patients, among whom 820 315 (52.0%) were male and 756 618 (48.0%) were female. For race and ethnicity, 74 369 (4.7%) were Asian American or Pacific Islander; 376 728 (23.9%), Hispanic; 267 056 (16.9%), non-Hispanic Black; 671 358 (42.6%), non-Hispanic White; 39 795 (2.5%), multiracial; and 147 627 (9.4%), other.

**Table 1.** Patient Characteristics

Characteristic	Patient cohort, No. (%)^a^		
	COVID-19–negative (n = 1 163 478)	COVID-19–positive (n = 413 455)	Overall (N = 1 576 933)
Age at cohort entry, y			
<1	132 283 (11.4)	57 135 (13.8)	189 418 (12.0)
1-4	349 078 (30.0)	105 599 (25.5)	454 677 (28.8)
5-11	366 863 (31.5)	121 287 (29.3)	488 150 (30.0)
12-18	315 254 (27.1)	129 434 (31.3)	444 688 (28.2)
Sex			
Female	554 659 (47.7)	201 959 (48.8)	756 618 (48.0)
Male	608 819 (52.3)	211 496 (51.2)	820 315 (52.0)
Race and ethnicity			
Asian American and Pacific Islander	54 636 (4.7)	19 733 (4.8)	74 369 (4.7)
Hispanic	274 391 (23.6)	102 337 (24.8)	376 728 (23.9)
Non-Hispanic Black	195 492 (16.8)	71 564 (17.3)	267 056 (16.9)
Non-Hispanic White	499 954 (43.0)	171 404 (41.5)	671 358 (42.6)
Multiracial	30 931 (2.7)	8864 (2.1)	39 795 (2.5)
Other^b^	108 074 (9.3)	39 553 (9.6)	147 627 (9.4)
Site index			
A	89 810 (7.7)	27 544 (6.7)	117 354 (7.4)
B	139 352 (12.0)	53 775 (13.0)	193 127 (12.2)
C	65 449 (5.6)	15 031 (3.6)	80 480 (5.1)
D	14 396 (1.2)	5102 (1.2)	19 498 (1.2)
E	39 273 (3.4)	8230 (2.0)	47 503 (3.0)
F	9426 (0.8)	4257 (1.0)	13 683 (0.9)
G	42 978 (3.7)	10 539 (2.5)	53 517 (3.4)
H	14 386 (1.2)	5743 (1.4)	20 129 (1.3)
I	28 785 (2.5)	9193 (2.2)	37 978 (2.4)
J	13 709 (1.2)	5054 (1.2)	18 763 (1.2)
K	80 676 (6.9)	36 989 (8.9)	117 665 (7.5)
L	105 681 (9.1)	22 904 (5.5)	128 585 (8.2)
M	43 508 (3.7)	13 741 (3.3)	57 249 (3.6)
N	3950 (0.3)	1553 (0.4)	5503 (0.3)
O	89 364 (7.7)	24 724 (6.0)	114 088 (7.2)
P	38 952 (3.3)	16 529 (4.0)	55 481 (3.5)
Q	30 009 (2.6)	8961 (2.2)	38 970 (2.5)
R	110 479 (9.5)	50 943 (12.3)	161 422 (10.2)
S	23 140 (2.0)	14 527 (3.5)	37 667 (2.4)
T	3339 (0.3)	1383 (0.3)	4722 (0.3)
U	12 724 (1.1)	23 894 (5.8)	36 618 (2.3)
V	15 556 (1.3)	4836 (1.2)	20 392 (1.3)
W	26 780 (2.3)	4831 (1.2)	31 611 (2.0)
X	38 333 (3.3)	12 499 (3.0)	50 832 (3.2)
Y	1039 (0.1)	772 (0.2)	1811 (0.1)
Z	35 262 (3.0)	11 130 (2.7)	46 392 (2.9)
AA	4719 (0.4)	2219 (0.5)	6938 (0.4)
AB	30 205 (2.6)	11 412 (2.8)	41 617 (2.6)
AC	12 198 (1.0)	5140 (1.2)	17 338 (1.1)
Cohort entry period			
March to May 2020	19 779 (1.7)	3793 (0.9)	23 572 (1.5)
June to August 2020	69 928 (6.0)	12 219 (3.0)	82 147 (5.2)
September to November 2020	98 028 (8.4)	20 709 (5.0)	118 737 (7.5)
December 2020 to February 2021	98 016 (8.4)	37 326 (9.0)	135 342 (8.6)
March to May 2021	107 170 (9.2)	22 859 (5.5)	130 029 (8.2)
June to August 2021	107 358 (9.2)	23 796 (5.8)	131 154 (8.3)
September to November 2021	162 532 (14.0)	38 181 (9.2)	200 713 (12.7)
December 2021 to February 2022	124 071 (10.7)	123 976 (30.0)	248 047 (15.7)
March to May 2022	93 889 (8.1)	31 854 (7.7)	125 743 (8.0)
June to August 2022	73 250 (6.3)	48 037 (11.6)	121 287 (7.7)
September to November 2022	113 927 (9.8)	24 082 (5.8)	138 009 (8.8)
December 2022 to March 2023	95 530 (8.2)	26 623 (6.4)	122 153 (7.7)
Obesity			
No	660 682 (56.8)	208 580 (50.4)	869 262 (55.1)
Yes	378 266 (32.5)	163 729 (39.6)	541 995 (34.4)
Unknown	124 530 (10.7)	41 146 (10.0)	165 676 (10.5)
PMCA index			
No chronic condition	811 758 (69.8)	289 146 (69.9)	1 100 904 (69.8)
Noncomplex chronic condition	193 920 (16.7)	69 616 (16.8)	263 536 (16.7)
Complex chronic condition	157 800 (13.6)	54 693 (13.2)	212 493 (13.5)
No. of tests			
0	856 237 (73.6)	245 524 (59.4)	1 101 761 (69.9)
1	193 189 (16.6)	87 927 (21.3)	281 116 (17.8)
2	62 803 (5.4)	37 458 (9.1)	100 261 (6.4)
≥3	51 249 (4.4)	42 546 (10.3)	93 795 (5.9)
No. of drugs			
0	278 662 (24.0)	80 867 (19.6)	359 529 (22.8)
1	137 488 (11.8)	45 771 (11.1)	183 259 (11.6)
2	111 410 (9.6)	40 194 (9.7)	151 604 (9.6)
≥3	635 918 (54.7)	246 623 (59.6)	882 541 (56.0)

Abbreviation: PMCA, Pediatric Medical Complexity Algorithm.

^a^ Percentages have been rounded and may not total 100.

^b^ Includes no information, refused to answer, or unknown categories.

**Figure 2.**
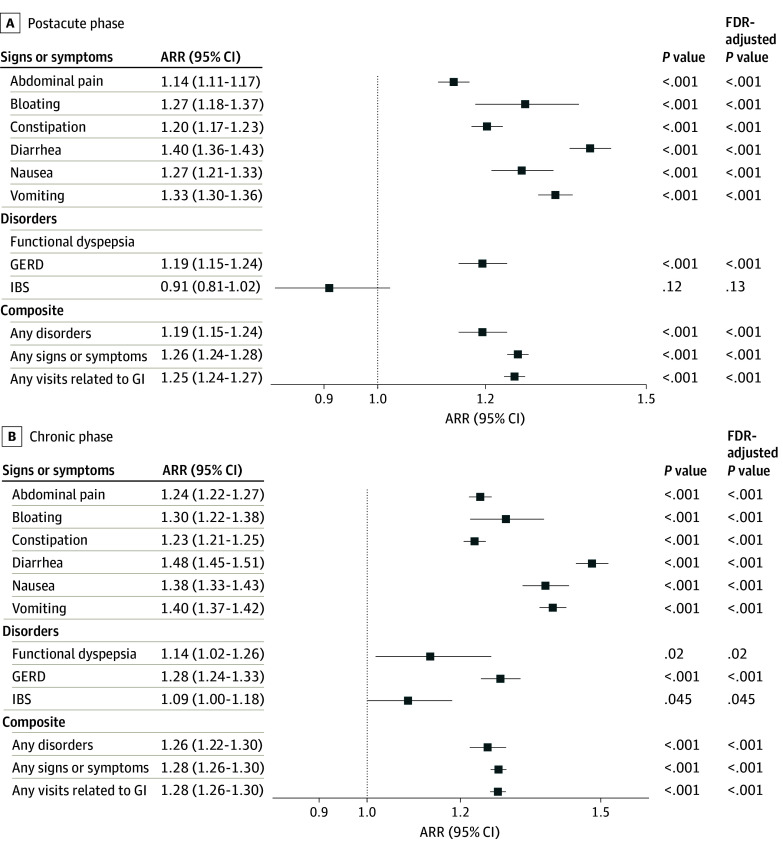
Adjusted Risk Ratios (ARRs) for Gastrointestinal (GI) Tract Outcomes in COVID-19–Positive vs –Negative Patients by Phase The outcomes are grouped by signs or symptoms, disorders, and composite outcomes. The postacute phase spans 28 to 179 days after cohort entry date, and the chronic phase spans 180 to 729 days after cohort entry date. The false discovery rate (FDR) is controlled by applying the Benjamini-Hochberg procedure. GERD indicates gastroesophageal reflux disease; IBS, irritable bowel syndrome.

The incidence of individual and composite GI tract conditions was higher among COVID-19–positive patients than among COVID-19–negative patients summarized in Table 2. We showed the ARR with 95% CI for each GI tract symptom and disorder and composite outcomes during the postacute phase or the chronic phase in Figure 2. The standardized mean differences of all baseline covariates were balanced (eFigures 2 and 3 in Supplement 1). We observed significantly elevated risks across various GI tract outcomes when comparing COVID-19–positive patients with their COVID-19–negative counterparts. Specifically, the risk of experiencing abdominal pain was higher in the COVID-19–positive group (2.54%) compared with the COVID-19–negative group (2.06%) in the postacute phase, with an ARR of 1.14 (95% CI, 1.11-1.17). Bloating was more prevalent in the COVID-19–positive group (0.28% vs 0.23%), with an ARR of 1.27 (95% CI, 1.18-1.37). Constipation showed an increased incidence (2.94% vs 2.42%), with an ARR of 1.20 (95% CI, 1.17-1.23). Diarrhea was reported more frequently (2.30% vs 1.57%), with an ARR of 1.40 (95% CI, 1.36-1.43). Incidences of nausea and vomiting were similarly elevated, with nausea at 0.81% vs 0.56% (ARR, 1.27; 95% CI, 1.21-1.33) and vomiting at 2.98% vs 2.29% (ARR, 1.33; 95% CI, 1.30-1.36). GERD also exhibited a higher incidence at 1.15% vs 1.00%, with an ARR of 1.19 (95% CI, 1.15-1.24). However, the risk of IBS was not statistically significant after adjusting for confounding variables (0.10% vs 0.11%; ARR, 0.91; 95% CI, 0.81-1.02). Additionally, the COVID-19–positive cohort exhibited increased risks for a composite of any of these GI tract signs or symptoms (8.24% vs 6.45%; ARR, 1.26; 95% CI, 1.24-1.28), any of these GI tract disorders (1.27% vs 1.12%; ARR, 1.19; 95% CI, 1.15-1.24), and any visits related to the GI tract (8.64% vs 6.85%; ARR, 1.25; 95% CI, 1.24-1.27).

**Table 2.** Incidence of Postacute or Chronic Phase by Documented COVID-19 Infection Status^a^

Outcome	No./total No. (%) of patients			
	Postacute phase		Chronic phase	
	COVID-19–positive	COVID-19–negative	COVID-19–positive	COVID-19–negative
Signs and symptoms				
Abdominal pain	9775/384 284 (2.54)	22 492/1 093 421 (2.06)	17 568/384 284 (4.57)	37 151/1 093 421 (3.40)
Bloating	1129/406 807 (0.28)	2595/1 148 213 (0.23)	1777/406 807 (0.44)	3900/1 148 213 (0.34)
Constipation	10 858/369 502 (2.94)	25 523/1 055 808 (2.42)	17 581/369 502 (4.76)	40 167/1 055 808 (3.80)
Diarrhea	8946/389 704 (2.30)	17 453/1 111 073 (1.57)	13 011/389 704 (3.34)	23 821/1 111 073 (2.14)
Nausea	3274/404 924 (0.81)	6398/1 145 467 (0.56)	5438/404 924 (1.34)	9802/1 145 467 (0.86)
Vomiting	11 290/378 336 (2.98)	24 673/1 078 123 (2.29)	16 064/378 336 (4.25)	32 852/1 078 123 (3.05)
Disorders				
GERD	4416/384 789 (1.15)	10 952/1 098 882 (1.00)	6132/384 789 (1.59)	13 336/1 098 882 (1.21)
IBS	419/412 180 (0.10)	1311/1 160 360 (0.11)	918/412 180 (0.22)	2210/1 160 360 (0.19)
Functional dyspepsia	325/412 490 (0.08)	789/1 161 209 (0.07)	548/412 490 (0.13)	1237/1 161 209 (0.11)
Composite				
Any signs or symptoms	25 810/313 153 (8.24)	59 218/918 811 (6.45)	38 168/313 153 (12.19)	83 894/918 811 (9.13)
Any disorders	4867/383 327 (1.27)	12 218/1 095 398 (1.12)	6962/383 327 (1.82)	15 380/1 095 398 (1.40)
Any visits related to GI tract	26 011/300 900 (8.64)	60 956/889 483 (6.85)	37 925/300 900 (12.60)	84 232/889 483 (9.47)

Abbreviations: GERD, gastroesophageal reflux disease; GI, gastrointestinal; IBS, irritable bowel syndrome.

^a^ Reported raw numbers and incidence for COVID-19–positive cohort (with documented COVID-19 infection) and COVID-19–negative cohort (without documented COVID-19 infection). The postacute phase is from 28 to 179 days after the cohort entry date; chronic phase, from 180 to 729 days after the cohort entry date. Incidence is defined as the number of patients who developed specified GI tract symptoms or disorders during the postacute or chronic phase, divided by the total at-risk population (those without these corresponding outcomes at the baseline period).

In the chronic phase shown in Figure 2, COVID-19–positive patients exhibited elevated risks of various GI tract symptoms and symptoms and disorders compared with the COVID-19–negative cohort. Specifically, the heightened risk persisted for abdominal pain (4.57% vs 3.40%; ARR, 1.24; 95% CI, 1.22-1.27) and for a composite of any of these GI tract signs or symptoms (12.19% vs 9.13%; ARR, 1.26; 95% CI, 1.22-1.30), any of these GI tract disorders (1.82% vs 1.40%; ARR, 1.28; 95% CI, 1.26-1.30), and any visits related to the GI tract (12.60% vs 9.47%; ARR, 1.28; 95% CI, 1.26-1.30). These findings highlighted the persistent higher health risks among individuals with documented COVID-19 across a range of GI tract outcomes for the chronic phase. Moreover, when compared with the postacute phase, the ARR for each GI tract outcome was higher in the chronic phase, indicating a prolonged increased risk of the documented infection on GI tract health.

**Figure 3.**
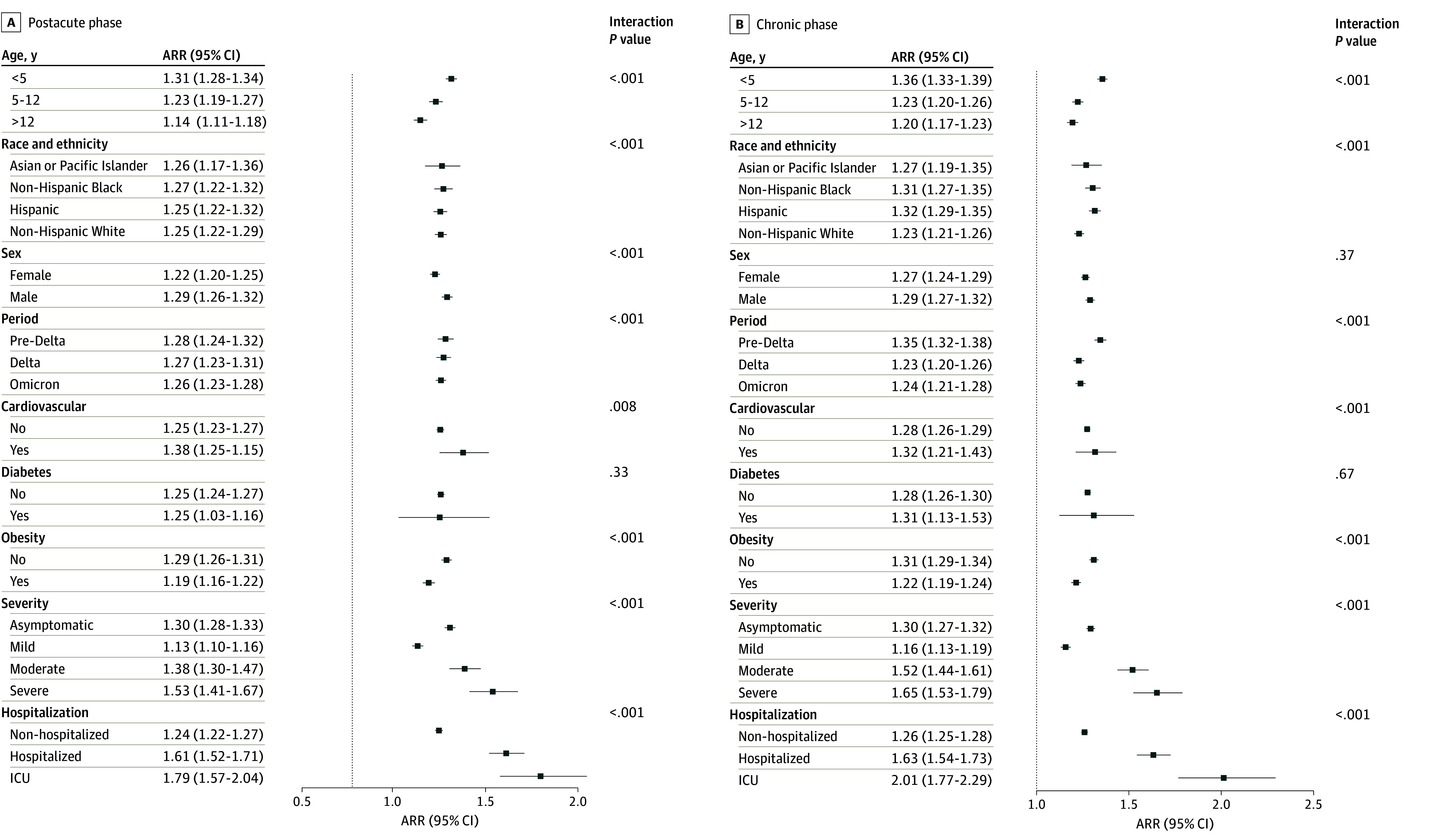
Subgroup Analysis of Adjusted Risk Ratios (ARR) for Gastrointestinal (GI) Tract Outcomes COVID-19–positive and –negative patients are compared across the postacute phase (28-179 days after cohort index date) and the chronic phase (180-729 days after cohort index date). Subgroups include age, race and ethnicity, sex, COVID-19 variant period, cardiovascular conditions, diabetes, obesity, severity of COVID-19, and hospitalization status. ICU indicates intensive care unit.

The results of the subgroup analysis for composite outcomes are shown in Figure 3, categorized by age, race and ethnicity, sex, cohort entry period, medical history of cardiovascular disease or diabetes, obesity status, the severity of the acute phase of COVID-19, and hospitalization status during the acute phase of COVID-19. Our findings revealed that children younger than 5 years were at the highest risk for GI tract disorders or symptoms compared with other age groups. Detailed results for the specific GI tract symptoms and disorders by age are shown in eTables 6 to 8 in Supplement 1. The ARR did not vary across different racial and ethnic groups, as presented in eTables 9 to 12 in Supplement 1. Interestingly, patients with obesity had a lower risk of GI tract symptoms or disorders compared with patients without obesity (eTables 13 and 14 in Supplement 1). Male patients exhibited a higher risk of GI tract disorders or symptoms during the postacute phase compared with female patients (eTables 15 and 16 in Supplement 1). No significant differences in the risk of GI tract symptoms and disorders were found between patients with and without diabetes (eTables 17 and 18 in Supplement 1) or between those with and without cardiovascular disease (eTables 19 and 20 in Supplement 1). However, the ARR of GI-related visits showed a progressive increase from the nonhospitalized group to the hospitalized group and to the group with intensive care unit admission group (eTables 21-23 in Supplement 1). Furthermore, the ARR of GI-related visits rose with the severity of the acute phase of COVID-19, escalating from the mild to the moderate and severe groups (eTables 24-27 in Supplement 1). Compared with the Omicron or Delta period, the risk of GI tract symptoms during the pre-Delta period was greater (eTables 28-30 in Supplement 1). We conducted negative control outcome experiments as a sensitivity analysis, which indicated a slight systematic bias (eFigures 4 and 5 in Supplement 1), evidenced by a minor shift in point estimates with wider CIs after the calibration (eTable 31 in Supplement 1). The minor systematic bias^34,35^ is likely attributable to unmeasured confounding, despite achieving a balance on observed covariates. This systematic bias suggests that residual confounding may persist in the estimate of ARRs.

The raw person-year incidence of GI tract symptoms or disorders during the chronic phase was higher in the COVID-19–positive group compared with the COVID-19–negative group (eTable 32 in Supplement 1). The increased risk of GI tract outcomes remained consistent after adding GI-related visits during the acute phase (eTable 33 in Supplement 1) or GI-related medications (eTable 34 in Supplement 1) as covariates in the propensity score model. Subgroup analyses showed a gradual increase in GI tract risk, from test positivity to COVID-19 diagnosis and then PASC diagnosis (eTables 35-37 in Supplement 1). eTable 38 in Supplement 1 presents the proportion of new GI tract symptoms or disorders among all GI tract symptoms or disorders.

## Discussion

In this population-based study of 1 576 933 US individuals 18 years or younger, we found a significantly increased risk of various GI tract outcomes associated with COVID-19, including abdominal pain, bloating, constipation, diarrhea, nausea, vomiting, and GERD. An outstanding feature of this study is its use of population-based databases within the RECOVER Initiative. This large program facilitated the precise identification of comprehensive documented COVID-19 infection records and visits associated with GI tract symptoms and disorders, unaffected by variations in testing practices since it exclusively includes cases from the 29 participating hospitals. Notably, our investigation extended into the chronic phase, with a follow-up period of up to 2 years, representing one of the longest follow-up durations reported for children and adolescents exposed to the documented COVID-19 infection in the US, to our knowledge.

Our findings align with prior research indicating that COVID-19 elevates the risk of GI tract symptoms such as nausea, diarrhea, and disorders like functional dyspepsia.^10,40,41,42,43,44^ Chronic GI tract symptoms following infections are well-documented in pediatric populations, often without a serious underlying cause.^45^ Our findings align with a pediatric study where symptoms such as abdominal pain, bloating, diarrhea, and nausea persisted in children for at least 6 months post infection.^46^ Additional postinfectious conditions—including constipation, vomiting and/or dyspepsia, and GERD—have also been observed in adults, underscoring the potential for infections to have long-term GI tract issues.^47,48^

One potential biological mechanism underlying this association is the high expression of angiotensin-converting enzyme 2 on the brush border of the small intestinal mucosa.^49^ Furthermore, SARS-CoV-2 infection has been shown to influence the gut microbiome,^50^ and the infection potentially persists beyond the postacute phase.^51^ Additionally, evidence from studies on prolonged viral fecal shedding^52^ and the persistence of the virus in the GI tract^53^ lends further support to the connection we have observed. A more profound understanding of the biological mechanisms will contribute to the development of targeted and effective interventions, ultimately improving outcomes for those affected by long-term GI tract disorders. Furthermore, from a clinical perspective, our findings emphasize the importance of considering the documented SARS-CoV-2 infection history when evaluating persistent GI tract symptoms in pediatric patients. Recognizing this association could reduce unnecessary testing or referrals, enabling clinicians to focus on timely and effective symptom management.

### Limitations

Our study has some limitations. First, while we successfully achieved a balanced distribution of baseline covariates through stratification, the propensity scores were constructed using only the variables accessible within our study databases. Minor systematic errors related to unmeasured confounding were identified using negative control outcomes. Despite our efforts to calibrate these biases, caution should be exercised when interpreting the results. Moreover, despite the large sample size, our study did not find associations for rare events such as IBS in the postacute phase. We excluded the analysis for the functional dyspepsia in the postacute phase analysis due to its rare occurrence (<0.1%), but we observed an increased ARR in functional dyspepsia during the chronic phase. Furthermore, while our findings indicate a nonsignificant risk reduction in the ARR for IBS during the postacute phase, it is important to note that the Rome IV criteria^54^ require a minimum of 6 months from symptom onset for a diagnosis of IBS. This time frame typically extends beyond the postacute period, complicating the diagnosis within that phase. Additionally, the misclassification of documented SARS-CoV-2 infection can be a problem within the EHR. However, recent studies^55^ provided a novel statistical method to account for the misclassified documented infection with prior knowledge about the prespecified range of the misclassification rates. Future research will seek to incorporate these advanced statistical techniques to account for potential misclassification of infection rates. Furthermore, while this study includes data from 29 US health institutions across urban and suburban regions, restricting the cohort to patients with regular access to pediatric academic centers may limit generalizability. Health-seeking behaviors, particularly among patients with GI tract symptoms or limited health care access, could lead to ascertainment bias in observed infection-related risks.

## Conclusions

In this cohort study of over 1.5 million US children and adolescents, we found a persistent association between documented SARS-CoV-2 infection and increased risks of GI tract symptoms, such as abdominal pain, bloating, constipation, and GERD, extending into the chronic phase. These findings underscore the potential for prolonged GI tract issues in pediatric COVID-19 cases, suggesting that a history of COVID-19 should be considered in evaluating persistent GI tract symptoms. They highlight the importance of ongoing monitoring for PASC outcomes in children and calls for further research to understand underlying mechanisms and improve targeted care.

## Notes

Supplement 1. **eAppendix 1.** Protocol for Analysis **eAppendix 2**. Names for Health Institutions **eAppendix 3**. Details for Primary Analysis and Code Availability **eAppendix 4**. Negative Control Outcome Experiments **eFigure 1.** Figure for Possible Mechanisms and Pathways for Patient Characteristics Variables **eFigure 2.** Standardized Mean Differences of Confounders for Outcomes During the Postacute Phase **eFigure 3.** Standardized Mean Differences of Confounders for Outcomes During the Chronic Phase **eFigure 4.** Estimated Systematic Bias During the Postacute Phase for the Subgroup of the Severe Group **eFigure 5.** Estimated Systematic Bias During the Chronic Phase for the Subgroup of the Severe Group **eTable 1.** *ICD-10-CM* Code Sets of GI Tract Outcomes **eTable 2.** *ICD-10-CM* Code Sets of Diabetes **eTable 3.** *ICD-10-CM* Code Sets of Cardiovascular Diseases **eTable 4.** Medication List for GI Tract Symptoms or Disorders **eTable 5.** List of Negative Control Outcomes **eTable 6.** Adjusted Risk Ratio for the Subgroup Younger Than 5 Years **eTable 7.** Adjusted Risk Ratio for the Subgroup Aged 5 to 11 Years **eTable 8.** Adjusted Risk Ratio for the Subgroup Aged 12 to 18 Years **eTable 9.** Adjusted Risk Ratio for the Non-Hispanic Black Subgroup **eTable 10.** Adjusted Risk Ratio for the Non-Hispanic White Subgroup **eTable 11.** Adjusted Risk Ratio for the Hispanic Subgroup **eTable 12.** Adjusted Risk Ratio for the Asian American and Pacific Islander Subgroup **eTable 13.** Adjusted Risk Ratio for the Subgroup With Obesity **eTable 14.** Adjusted Risk Ratio for the Subgroup Without Obesity **eTable 15.** Adjusted Risk Ratio for the Female Subgroup **eTable 16.** Adjusted Risk Ratio for the Male Subgroup **eTable 17.** Adjusted Risk Ratio for the Subgroup With a Medical History of Diabetes **eTable 18.** Adjusted Risk Ratio for the Subgroup Without a Medical History of Diabetes **eTable 19.** Adjusted Risk Ratio for the Subgroup With a Medical History of Cardiovascular Diseases **eTable 20.** Adjusted Risk Ratio for the Subgroup Without a Medical History of Cardiovascular Diseases **eTable 21.** Adjusted Risk Ratio for the Subgroup Without Hospitalization **eTable 22.** Adjusted Risk Ratio for the Subgroup With Hospitalization **eTable 23.** Adjusted Risk Ratio for the Subgroup With ICU Admission **eTable 24.** Adjusted Risk Ratio for the Asymptomatic Subgroup **eTable 25.** Adjusted Risk Ratio for the Mild Subgroup **eTable 26.** Adjusted Risk Ratio for the Moderate Subgroup **eTable 27.** Adjusted Risk Ratio for the Severe Subgroup **eTable 28.** Adjusted Risk Ratio for the Pre-Delta Subgroup **eTable 29.** Adjusted Risk Ratio for the Delta Subgroup **eTable 30.** Adjusted Risk Ratio for the Omicron Subgroup **eTable 31.** Calibrated Risk Ratios for the GI Tract Outcomes During the Postacute or Chronic Phase **eTable 32.** Incidence by Person-Years for Chronic Phase by Documented COVID-19 Infection Status **eTable 33.** Adjusted Risk Ratios Adding GI-Related Visits During the Acute Phase to Propensity Score **eTable 34.** Adjusted Risk Ratios Adding GI-Related Medications Before Postacute Phase to Propensity Score **eTable 35.** Adjusted Risk Ratios for the Test Positivity Subgroup **eTable 36.** Adjusted Risk Ratios for the COVID-19 Diagnosis Subgroup **eTable 37.** Adjusted Risk Ratios for the PASC Diagnosis Subgroup **eTable 38.** Relative Proportion of GI Tract Signs, Symptoms and Disorders to Overall GI Outcomes by Documented SARS-CoV-2 Infection Status
eReferences.

Supplement 2. **Nonauthor Collaborators.** RECOVER Initiative Members

Supplement 3.
Data Sharing Statement
