# Supplementary material for: Pediatric Gastrointestinal Tract Outcomes During the Postacute Phase of COVID-19
Source: JAMA Netw Open. 2025 Feb 7;8(2):e2458366. doi: 10.1001/jamanetworkopen.2024.58366 (PMC11806396; doi:10.1001/jamanetworkopen.2024.58366)
Supplement: Supplement 1. — eAppendix 1. Protocol for Analysis eAppendix 2. Names for Health Institutions eAppendix 3. Details for Primary Analysis and Code Availability eAppendix 4. Negative Control Outcome Experiments eFigure 1. Figure for Possible Mechanisms and Pathways for Patient Characteristics Variables eFigure 2. Standardized Mean Differences of Confounders for Outcomes During the Postacute Phase eFigure 3. Standardized Mean Differences of Confounders for Outcomes During the Chronic Phase eFigure 4. Estimated Systematic Bias During the Postacute Phase for the Subgroup of the Severe Group eFigure 5. Estimated Systematic Bias During the Chronic Phase for the Subgroup of the Severe Group eTable 1. ICD-10-CM Code Sets of GI Tract Outcomes eTable 2. ICD-10-CM Code Sets of Diabetes eTable 3. ICD-10-CM Code Sets of Cardiovascular Diseases eTable 4. Medication List for GI Tract Symptoms or Disorders eTable 5. List of Negative Control Outcomes eTable 6. Adjusted Risk Ratio for the Subgroup Younger Than 5 Years eTable 7. Adjusted Risk Ratio for the Subgroup Aged 5 to 11 Years eTable 8. Adjusted Risk Ratio for the Subgroup Aged 12 to 18 Years eTable 9. Adjusted Risk Ratio for the Non-Hispanic Black Subgroup eTable 10. Adjusted Risk Ratio for the Non-Hispanic White Subgroup eTable 11. Adjusted Risk Ratio for the Hispanic Subgroup eTable 12. Adjusted Risk Ratio for the Asian American and Pacific Islander Subgroup eTable 13. Adjusted Risk Ratio for the Subgroup With Obesity eTable 14. Adjusted Risk Ratio for the Subgroup Without Obesity eTable 15. Adjusted Risk Ratio for the Female Subgroup eTable 16. Adjusted Risk Ratio for the Male Subgroup eTable 17. Adjusted Risk Ratio for the Subgroup With a Medical History of Diabetes eTable 18. Adjusted Risk Ratio for the Subgroup Without a Medical History of Diabetes eTable 19. Adjusted Risk Ratio for the Subgroup With a Medical History of Cardiovascular Diseases eTable 20. Adjusted Risk Ratio for the Subgroup Without a Medical History of Cardiovascular Dis [file jamanetwopen-e2458366-s001.pdf]

Supplemental Online Content

Zhang D, Stein R, Lu Y, et al; Researching COVID to Enhance Recovery Initiative. Pediatric gastrointestinal tract outcomes during the postacute phase of COVID-19. *JAMA Network Open*. 2025;8(2):e2458366. doi:10.1001/jamanetworkopen.2024.58366

eAppendix 1. Protocol for Analysis
eAppendix 2. Names for Health Institutions
eAppendix 3. Details for Primary Analysis and Code Availability
eAppendix 4. Negative Control Outcome Experiments
eFigure 1. Figure for Possible Mechanisms and Pathways for Patient Characteristics Variables
eFigure 2. Standardized Mean Differences of Confounders for Outcomes During the Postacute Phase
eFigure 3. Standardized Mean Differences of Confounders for Outcomes During the Chronic Phase
eFigure 4. Estimated Systematic Bias During the Postacute Phase for the Subgroup of the Severe Group
eFigure 5. Estimated Systematic Bias During the Chronic Phase for the Subgroup of the Severe Group
eTable 1. *ICD-10-CM* Code Sets of GI Tract Outcomes
eTable 2. *ICD-10-CM* Code Sets of Diabetes
eTable 3. *ICD-10-CM* Code Sets of Cardiovascular Diseases
eTable 4. Medication List for GI Tract Symptoms or Disorders
eTable 5. List of Negative Control Outcomes
eTable 6. Adjusted Risk Ratio for the Subgroup Younger Than 5 Years
eTable 7. Adjusted Risk Ratio for the Subgroup Aged 5 to 11 Years
eTable 8. Adjusted Risk Ratio for the Subgroup Aged 12 to 18 Years
eTable 9. Adjusted Risk Ratio for the Non-Hispanic Black Subgroup
eTable 10. Adjusted Risk Ratio for the Non-Hispanic White Subgroup
eTable 11. Adjusted Risk Ratio for the Hispanic Subgroup
eTable 12. Adjusted Risk Ratio for the Asian American and Pacific Islander Subgroup
eTable 13. Adjusted Risk Ratio for the Subgroup With Obesity
eTable 14. Adjusted Risk Ratio for the Subgroup Without Obesity
eTable 15. Adjusted Risk Ratio for the Female Subgroup
eTable 16. Adjusted Risk Ratio for the Male Subgroup
eTable 17. Adjusted Risk Ratio for the Subgroup With a Medical History of Diabetes
eTable 18. Adjusted Risk Ratio for the Subgroup Without a Medical History of Diabetes
eTable 19. Adjusted Risk Ratio for the Subgroup With a Medical History of Cardiovascular Diseases
eTable 20. Adjusted Risk Ratio for the Subgroup Without a Medical History of Cardiovascular Diseases
eTable 21. Adjusted Risk Ratio for the Subgroup Without Hospitalization
eTable 22. Adjusted Risk Ratio for the Subgroup With Hospitalization
eTable 23. Adjusted Risk Ratio for the Subgroup With ICU Admission
eTable 24. Adjusted Risk Ratio for the Asymptomatic Subgroup
eTable 25. Adjusted Risk Ratio for the Mild Subgroup
eTable 26. Adjusted Risk Ratio for the Moderate Subgroup
eTable 27. Adjusted Risk Ratio for the Severe Subgroup
eTable 28. Adjusted Risk Ratio for the Pre-Delta Subgroup
eTable 29. Adjusted Risk Ratio for the Delta Subgroup
eTable 30. Adjusted Risk Ratio for the Omicron Subgroup
eTable 31. Calibrated Risk Ratios for the GI Tract Outcomes During the Postacute or Chronic Phase
eTable 32. Incidence by Person-Years for Chronic Phase by Documented COVID-19 Infection Status
eTable 33. Adjusted Risk Ratios Adding GI-Related Visits During the Acute Phase to Propensity Score
eTable 34. Adjusted Risk Ratios Adding GI-Related Medications Before Postacute Phase to Propensity Score
eTable 35. Adjusted Risk Ratios for the Test Positivity Subgroup
eTable 36. Adjusted Risk Ratios for the COVID-19 Diagnosis Subgroup
eTable 37. Adjusted Risk Ratios for the PASC Diagnosis Subgroup

© 2025 Zhang D et al. *JAMA Network Open*.

eTable 38. Relative Proportion of GI Tract Signs, Symptoms and Disorders to Overall GI Outcomes by Documented SARS-CoV-2 Infection Status
eReferences

This supplementary material has been provided by the authors to give readers additional information about their work.

© 2025 Zhang D et al. *JAMA Network Open*.

eAppendix 1. Protocol for Analysis

To enhance the reproducibility and transparency of our research findings, we pre-specify our statistical analysis plan and protocol as follows. This ensures that our methods are clearly defined and adhered to throughout the study, promoting the reliability and replicability of our results. We believe this commitment to methodological transparency strengthens the impact and credibility of our research within the scientific community.

Aims

- To evaluate the risk of gastrointestinal outcomes after the infection of SARS-CoV-2 during the post-acute phase (days 28 to 179) for children and adolescents.
- To evaluate the risk of gastrointestinal outcomes after the infection of SARS-CoV-2 during the chronic phase (days 180 to 729) for children and adolescents.

Design

- COVID-19 positive cohort
  - Selection criteria
    - Infection of COVID-19 during 2020-03-01~2023-03-06: PCR/antigen/serology/covid diagnosis (specific, complication, history)/PASC:
      - Index date: earliest date of (COVID-19 infection) or (PASC) diagnosis - 28 days).
    - Satisfy the age constraint (exclude those above 18).
    - Had at least one visit 28 - 179 days after the index date.
    - Require at least one prior visit: -24 months ~ -7 days.
    - Require at least one visit during follow-up: 28 ~ 179 days.
    - Exclude MISC patients.
- Contemporary control cohort:
  - Selection criteria:
    - At least a negative test of COVID-19 during 2020-03-01~2023-03-06: PCR/antigen/serology:
      - No evidence of COVID-19.
      - All virus tests are negative and no COVID diagnosis/PASC/MISC.
      - Index date: a randomly selected negative test date.
    - Satisfy the age constraint at the index date (exclude those above 18).
    - Require at least one prior visit: -24 months ~ -7 days.
    - Require at least one visit during follow-up: 28 ~ 179 days.

Variables

- Exposure: Documented SARS-CoV-2 infection or not
- Outcomes

**Disorders**	IBS
	GERD

© 2025 Zhang D et al. *JAMA Network Open*.

	Functional dyspepsia
**Signs and symptoms**	Constipation
	Abdominal pain
	Diarrhea
	Vomiting
	Bloating
	Nausea

- Three composite outcomes:
  - Any GI disorders.
  - Any signs and symptoms of GI.
  - Any visits related to GI.

- Confounders
  - Demographics (age at entry; race/ethnicity; sex)
  - Obesity status
  - PMCA
  - Healthcare utilization prior to index (categorized to 0,1,2,>2):
    - Number of outpatient visits (2 years ~ 7 days prior to index date).
    - Number of inpatient visits (2 years ~ 7 days prior to index date).
    - Number of ED visits (2 years ~ 7 days prior to index date).
    - Number of unique medications/prescriptions (2 years ~ 7 days prior to index date).
  - The cohort entry date is categorized as 1 month.
  - Hospital index (site).
  - Medical history: List of chronic conditions (2 years ~ 7 days before the index date).

- Negative control outcomes:
  - 36 negative control outcomes were evaluated in the same post-acute period and chronic period.

Statistical analysis

- Calculate the incidence of each gastrointestinal outcome and composite outcomes.
  - The nominator of the incidence of the GI outcome is the number of new cases during the post-acute phase/chronic phase.
  - The denominator of the incidence of the GI outcome is the number of individuals at risk during the post-acute phase/chronic phase.

- Risks of the incident of gastrointestinal outcomes comparing COVID-19 positive cohort with the contemporary control cohort during the **post-acute phase** while adjusting for observed confounders.
    - Fit logistic regression model to get the propensity score (COVID-19 vs. contemporary).
    - Evaluate confounders' balance by SMD, using 0.1 as the cut-off value.
    - To balance confounders distribution between the COVID-19 positive cohort and the contemporary control cohort, we use stratification by propensity score.
    - Fit stratified Poisson model to estimate hazard ratios (risk ratios) during the post-acute phase.
- Risks of the incident of gastrointestinal outcomes comparing COVID-19 positive cohort with contemporary control cohort during **chronic phase** while adjusting for observed confounders.
    - Fit logistic regression model to get the propensity score (COVID-19 vs. contemporary).
    - Evaluate the confounders' balance by SMD, using 0.1 as the cut-off value.
    - To balance confounders distribution between the COVID-19 positive cohort and the contemporary control cohort, we use stratification by propensity score.
    - Fit stratified Poisson model to estimate hazard ratios (risk ratios) during the chronic phase with offset for person-year.

Sensitivity analysis

- Subgroup analysis by risk factors (age/race/sex/period/obesity/cardiovascular/diabetes).
- They were stratified by severity during the acute phase of SARS-CoV-2 infection.
    - 4 levels of severity.
- Hospitalization.
- Negative control calibration.
- Add potential confounders separately to the propensity score model: GI-visits during the acute-phase and GI-related medications before the post-acute phase.
- Different types of index date (COVID-19 diagnosis, PASC diagnosis, and Test positivity).
- Incidence for person-year for chronic phase.
- Proportion of new symptoms or disorders among all symptoms or disorders.

eAppendix 2. Names for Health Institutions

The twenty-nine health institutions included Children's Hospital of Philadelphia, Cincinnati Children's Hospital Medical Center, Children's Hospital Colorado, Columbia University Irving Medical Center, Duke University, The Ohio State University, Ann & Robert H. Lurie Children's Hospital of Chicago, Medical College of Wisconsin, University of Michigan, University of Missouri, Montefiore, Medical University of South Carolina, Children's National Medical Center, Nationwide Children's Hospital, Nicklaus Children's Hospital, the University of Nebraska Medical Center, The Nemours Foundation, Northwestern University, New York University School of Medicine, OCHIN, Inc., Ochsner Health System, University of Pittsburgh, Penn State University, Seattle Children's Hospital, Stanford University, University of California San Francisco, University of South Florida and Tampa, Vanderbilt University Medical Center, and Weill Cornell Medical College.

eAppendix 3. Details for Primary Analysis and Code Availability

We divided the study population into six strata based on the propensity score distribution, ensuring that each stratum represented a distinct risk profile. Within each stratum, adjusted relative risks (aRR) were estimated using a modified Poisson regression model. To calculate an overall aRR across strata, we combined estimates from each stratum using a weighted average approach, where the weight for each stratum was proportional to the number of participants within it. This method ensures that the overall estimate reflects the distribution of the study population,

© 2025 Zhang D et al. *JAMA Network Open*.

improving the accuracy and interpretability of the aRR. Additionally, we provide a link to our GitHub repository as follows: https://github.com/Penncil/Long_term_GI.

eAppendix 4. Negative Control Outcome Experiments

We conducted negative control outcome experiments[1,2] to adjust for the systematic bias potentially introduced by unmeasured confounders. Specifically, we employed a Poisson model using thirty-six predetermined negative controls and covariates, along with the infection status and the confounders detailed in **Table 1** of the main manuscript. The adjusted risk ratios of these negative control outcomes indicated the potential systematic bias attributable to unmeasured confounders. We then calculated the systematic bias from these adjusted risk ratios and used the estimates to recalibrate the adjusted risk ratios presented in **Figure 2** of the main manuscript. The results are as follows.

© 2025 Zhang D et al. *JAMA Network Open*.

eFigure 1. Figure for Possible Mechanisms and Pathways for Patient Characteristics Variables

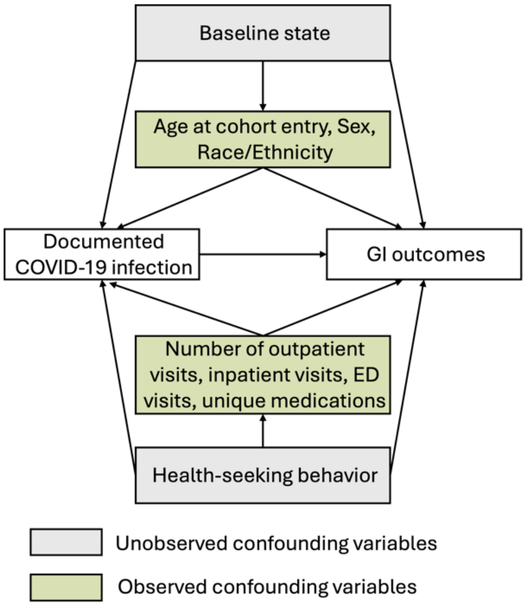

eFigure 2. Standardized Mean Differences of Confounders for Outcomes During the Post-acute Phase

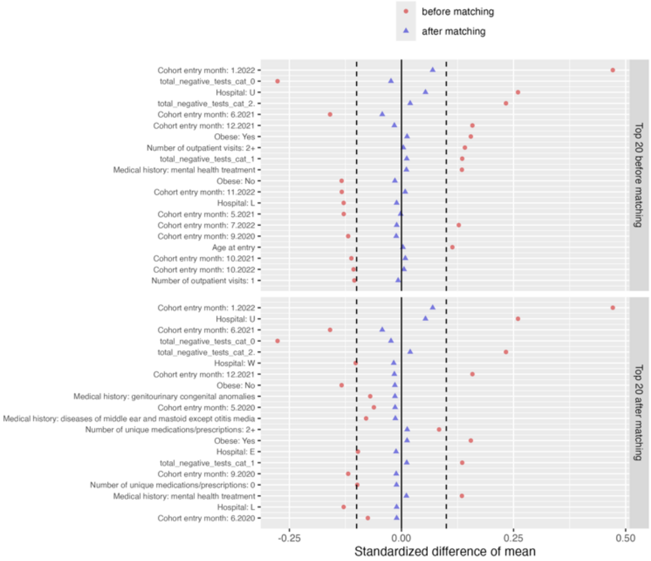

© 2025 Zhang D et al. *JAMA Network Open*.

eFigure 3. Standardized Mean Differences of Confounders for Outcomes During the Chronic Phase

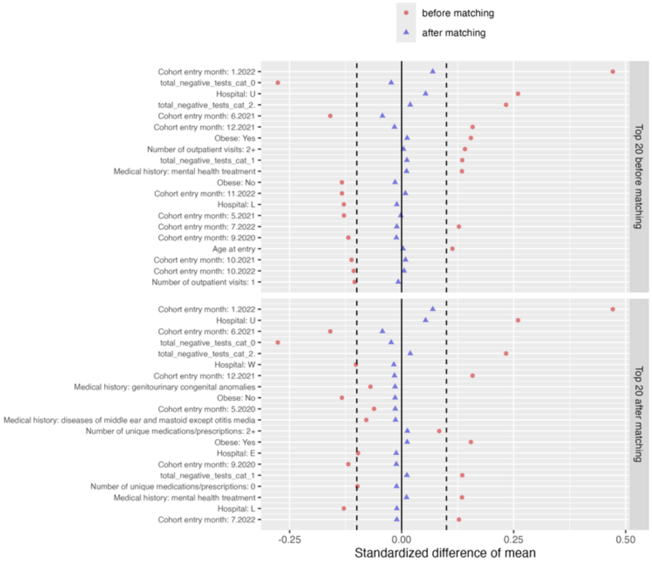

© 2025 Zhang D et al. *JAMA Network Open*.

eFigure 4. Estimated Systematic Bias During the Postacute Phase for the Subgroup of the Severe Group

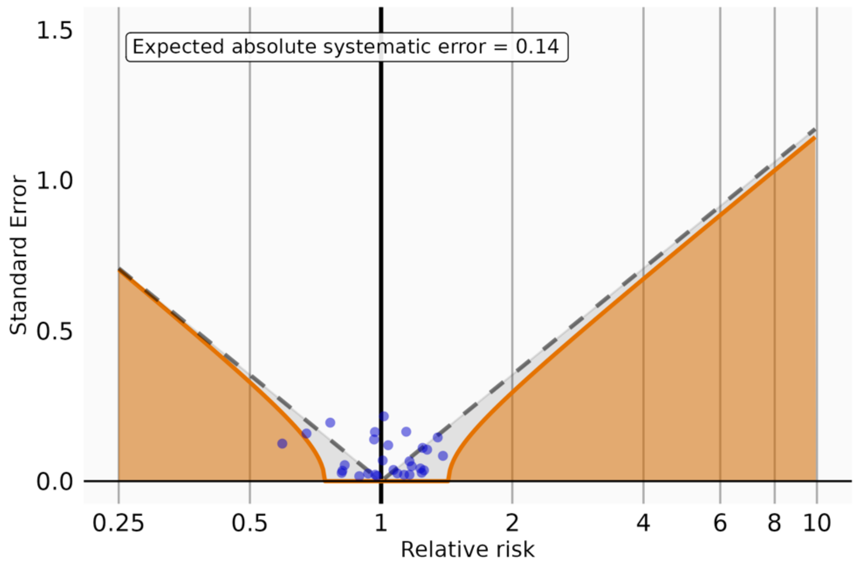

eFigure 5. Estimated Systematic Bias During the Chronic Phase for the Subgroup of the Severe Group

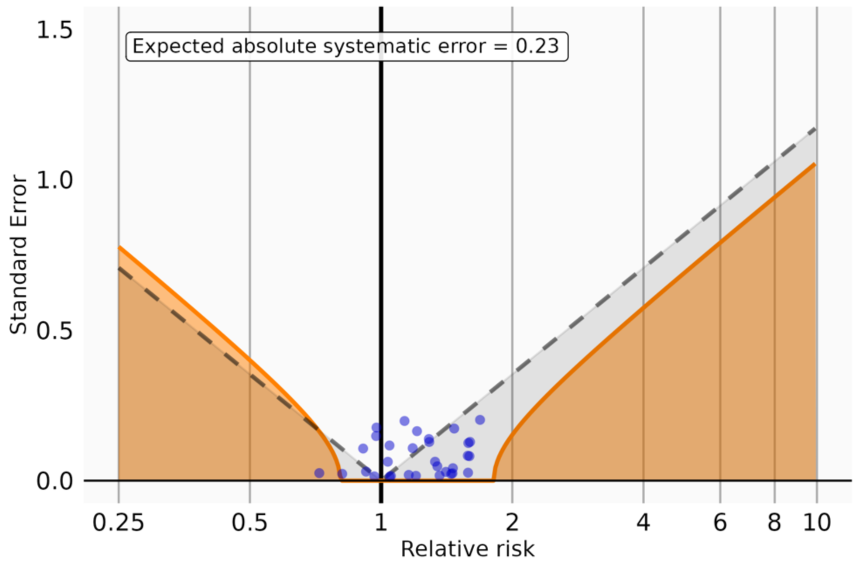

© 2025 Zhang D et al. *JAMA Network Open*.

eTable 1. *ICD-10-CM* Code Sets of GI Tract Outcomes

Concept id	Concept name	GI outcomes	ICD10-CM code
**45577781**	Upper abdominal pain, unspecified	Abdominal pain	R10.10
**45563290**	Lower abdominal pain, unspecified	Abdominal pain	R10.30
**1572201**	Other abdominal pain	Abdominal pain	R10.8
**45558455**	Generalized abdominal pain	Abdominal pain	R10.84
**45568114**	Unspecified abdominal pain	Abdominal pain	R10.9
**1572205**	Flatulence and related conditions	Bloating	R14
**45582697**	Abdominal distension (gaseous)	Bloating	R14.0
**45592410**	Gas pain	Bloating	R14.1
**45539321**	Flatulence	Bloating	R14.3
**1569650**	Constipation	Constipation	K59.0
**45601182**	Constipation, unspecified	Constipation	K59.00
**45557664**	Slow transit constipation	Constipation	K59.01
**45572219**	Outlet dysfunction constipation	Constipation	K59.02
**37200649**	Drug induced constipation	Constipation	K59.03
**37200650**	Chronic idiopathic constipation	Constipation	K59.04
**45552941**	Other constipation	Constipation	K59.09
**35208288**	Functional diarrhea	Diarrhea	K59.1
**35210644**	Noninfective neonatal diarrhea	Diarrhea	P78.3
**45534435**	Diarrhea, unspecified	Diarrhea	R19.7
**35208237**	Functional dyspepsia	Functional dyspepsia	K30

© 2025 Zhang D et al. *JAMA Network Open*.

**1569558**	Gastro-esophageal reflux disease	GERD	K21
**35208189**	Gastro-esophageal reflux disease with esophagitis	GERD	K21.0
**725363**	Gastro-esophageal reflux disease with esophagitis, without bleeding	GERD	K21.00
**725364**	Gastro-esophageal reflux disease with esophagitis, with bleeding	GERD	K21.01
**35208190**	Gastro-esophageal reflux disease without esophagitis	GERD	K21.9
**35209247**	Nonobstructive reflux-associated chronic pyelonephritis	GERD	N11.0
**1571478**	Obstructive and reflux uropathy	GERD	N13
**45592347**	Newborn esophageal reflux	GERD	P78.83
**1569648**	Irritable bowel syndrome	IBS	K58
**35208286**	Irritable bowel syndrome with diarrhea	IBS	K58.0
**37200646**	Irritable bowel syndrome with constipation	IBS	K58.1
**37200647**	Mixed irritable bowel syndrome	IBS	K58.2
**37200648**	Other irritable bowel syndrome	IBS	K58.8
**35208287**	Irritable bowel syndrome without diarrhea	IBS	K58.9
**45534429**	Nausea	Nausea	R11.0

© 2025 Zhang D et al. *JAMA Network Open*.

**45581492**	Cyclical vomiting	Vomiting	G43.A
**45538112**	Cyclical vomiting, in migraine, not intractable	Vomiting	G43.A0
**45605547**	Cyclical vomiting, in migraine, intractable	Vomiting	G43.A1
**35210670**	Vomiting of newborn	Vomiting	P92.0
**45606715**	Bilious vomiting of newborn	Vomiting	P92.01
**45553654**	Other vomiting of newborn	Vomiting	P92.09
**45534430**	Vomiting	Vomiting	R11.1
**45602008**	Vomiting, unspecified	Vomiting	R11.10
**45573011**	Vomiting without nausea	Vomiting	R11.11
**45558456**	Projectile vomiting	Vomiting	R11.12
**45573012**	Vomiting of fecal matter	Vomiting	R11.13
**45568115**	Bilious vomiting	Vomiting	R11.14
**1553844**	Cyclical vomiting syndrome unrelated to migraine	Vomiting	R11.15
**35208059**	Pneumonitis due to inhalation of food and vomit	Vomiting	J69.0

eTable 2. *ICD-10-CM* Code Sets of Diabetes

**Concept id**	**Concept name**	**ICD10-CM code**
**1567906**	Diabetes mellitus due to underlying condition	E08
**1567907**	Diabetes mellitus due to underlying condition with hyperosmolarity	E08.0
**1567908**	Diabetes mellitus due to underlying condition with ketoacidosis	E08.1
**1567909**	Diabetes mellitus due to underlying condition with kidney complications	E08.2
**1567910**	Diabetes mellitus due to underlying condition with ophthalmic complications	E08.3
**1567916**	Diabetes mellitus due to underlying condition with neurological complications	E08.4
**1567917**	Diabetes mellitus due to underlying condition with circulatory complications	E08.5
**1567918**	Diabetes mellitus due to underlying condition with other specified complications	E08.6
**45605395**	Diabetes mellitus due to underlying condition with unspecified complications	E08.8
**45571652**	Diabetes mellitus due to underlying condition without complications	E08.9
**1567925**	Drug or chemical induced diabetes mellitus with ketoacidosis	E09.1

© 2025 Zhang D et al. *JAMA Network Open*.

**1567926**	Drug or chemical induced diabetes mellitus with kidney complications	E09.2
**1567927**	Drug or chemical induced diabetes mellitus with ophthalmic complications	E09.3
**1567933**	Drug or chemical induced diabetes mellitus with neurological complications	E09.4
**1567934**	Drug or chemical induced diabetes mellitus with circulatory complications	E09.5
**1567935**	Drug or chemical induced diabetes mellitus with other specified complications	E09.6
**45586136**	Drug or chemical induced diabetes mellitus with unspecified complications	E09.8
**45533015**	Drug or chemical induced diabetes mellitus without complications	E09.9
**1567941**	Type 1 diabetes mellitus with ketoacidosis	E10.1
**1567973**	Other specified diabetes mellitus with hyperosmolarity	E13.0
**1567974**	Other specified diabetes mellitus with ketoacidosis	E13.1
**1567975**	Other specified diabetes mellitus with kidney complications	E13.2
**1567976**	Other specified diabetes mellitus with ophthalmic complications	E13.3
**1567982**	Other specified diabetes mellitus with neurological complications	E13.4
**1567983**	Other specified diabetes mellitus with circulatory complications	E13.5
**1567984**	Other specified diabetes mellitus with other specified complications	E13.6
**35206884**	Other specified diabetes mellitus with unspecified complications	E13.8
**35206885**	Other specified diabetes mellitus without complications	E13.9
**35207109**	Postprocedural hypoinsulinemia	E89.1
**45606548**	Unspecified pre-existing diabetes mellitus in childbirth	O24.32
**45534189**	Unspecified pre-existing diabetes mellitus in the puerperium	O24.33
**45534190**	Other pre-existing diabetes mellitus in childbirth	O24.82
**45592199**	Other pre-existing diabetes mellitus in the puerperium	O24.83
**1567942**	Type 1 diabetes mellitus with kidney complications	E10.2
**1567943**	Type 1 diabetes mellitus with ophthalmic complications	E10.3
**1567949**	Type 1 diabetes mellitus with neurological complications	E10.4
**1567950**	Type 1 diabetes mellitus with circulatory complications	E10.5
**1567951**	Type 1 diabetes mellitus with other specified complications	E10.6
**35206878**	Type 1 diabetes mellitus with unspecified complications	E10.8
**35206879**	Type 1 diabetes mellitus without complications	E10.9
**45606546**	Pre-existing type 1 diabetes mellitus, in childbirth	O24.02
**45577566**	Pre-existing type 1 diabetes mellitus, in the puerperium	O24.03
**1326491**	Type 2 diabetes mellitus with ketoacidosis	E11.1
**1567958**	Type 2 diabetes mellitus with kidney complications	E11.2
**1567959**	Type 2 diabetes mellitus with ophthalmic complications	E11.3
**1567965**	Type 2 diabetes mellitus with neurological complications	E11.4
**1567966**	Type 2 diabetes mellitus with circulatory complications	E11.5

© 2025 Zhang D et al. *JAMA Network Open*.

**1567967**	Type 2 diabetes mellitus with other specified complications	E11.6
**35206881**	Type 2 diabetes mellitus with unspecified complications	E11.8
**35206882**	Type 2 diabetes mellitus without complications	E11.9
**45587293**	Pre-existing type 2 diabetes mellitus, in childbirth	O24.12
**45582458**	Pre-existing type 2 diabetes mellitus, in the puerperium	O24.13

eTable 3. *ICD-10-CM* Code Sets of Cardiovascular Diseases

Concept id	Concept name	ICD10-CM code
**35207393**	Vertebro-basilar artery syndrome	G45.0
**35207394**	Carotid artery syndrome (hemispheric)	G45.1
**35207395**	Multiple and bilateral precerebral artery syndromes	G45.2
**35207396**	Amaurosis fugax	G45.3
**35207397**	Transient global amnesia	G45.4
**35207398**	Other transient cerebral ischemic attacks and related syndromes	G45.8
**35207399**	Transient cerebral ischemic attack, unspecified	G45.9
**45605800**	Nontraumatic subarachnoid hemorrhage from unspecified carotid siphon and bifurcation	I60.00
**45533459**	Nontraumatic subarachnoid hemorrhage from right carotid siphon and bifurcation	I60.01
**45605801**	Nontraumatic subarachnoid hemorrhage from left carotid siphon and bifurcation	I60.02
**45538388**	Nontraumatic subarachnoid hemorrhage from unspecified middle cerebral artery	I60.10
**45552799**	Nontraumatic subarachnoid hemorrhage from right middle cerebral artery	I60.11
**45533460**	Nontraumatic subarachnoid hemorrhage from left middle cerebral artery	I60.12
**1569187**	Nontraumatic subarachnoid hemorrhage from anterior communicating artery	I60.2
**45591471**	Nontraumatic subarachnoid hemorrhage from unspecified anterior communicating artery	I60.20
**45562357**	Nontraumatic subarachnoid hemorrhage from right anterior communicating artery	I60.21
**45605802**	Nontraumatic subarachnoid hemorrhage from left anterior communicating artery	I60.22
**45538389**	Nontraumatic subarachnoid hemorrhage from unspecified posterior communicating artery	I60.30
**45538390**	Nontraumatic subarachnoid hemorrhage from right posterior communicating artery	I60.31
**45562359**	Nontraumatic subarachnoid hemorrhage from left posterior communicating artery	I60.32
**35207803**	Nontraumatic subarachnoid hemorrhage from basilar artery	I60.4
**45586591**	Nontraumatic subarachnoid hemorrhage from unspecified vertebral artery	I60.50
**45567185**	Nontraumatic subarachnoid hemorrhage from right vertebral artery	I60.51
**45543185**	Nontraumatic subarachnoid hemorrhage from left vertebral artery	I60.52

© 2025 Zhang D et al. *JAMA Network Open*.

**35207804**	Nontraumatic subarachnoid hemorrhage from other intracranial arteries	I60.6
**35207805**	Nontraumatic subarachnoid hemorrhage from unspecified intracranial artery	I60.7
**35207806**	Other nontraumatic subarachnoid hemorrhage	I60.8
**35207807**	Nontraumatic subarachnoid hemorrhage, unspecified	I60.9
**1569190**	Nontraumatic intracerebral hemorrhage	I61
**35207808**	Nontraumatic intracerebral hemorrhage in hemisphere, subcortical	I61.0
**35207809**	Nontraumatic intracerebral hemorrhage in hemisphere, cortical	I61.1
**35207810**	Nontraumatic intracerebral hemorrhage in hemisphere, unspecified	I61.2
**35207811**	Nontraumatic intracerebral hemorrhage in brain stem	I61.3
**35207812**	Nontraumatic intracerebral hemorrhage in cerebellum	I61.4
**35207813**	Nontraumatic intracerebral hemorrhage, intraventricular	I61.5
**35207814**	Nontraumatic intracerebral hemorrhage, multiple localized	I61.6
**35207815**	Other nontraumatic intracerebral hemorrhage	I61.8
**35207816**	Nontraumatic intracerebral hemorrhage, unspecified	I61.9
**45538392**	Nontraumatic subdural hemorrhage, unspecified	I62.00
**45557548**	Nontraumatic acute subdural hemorrhage	I62.01
**45605805**	Nontraumatic subacute subdural hemorrhage	I62.02
**45557549**	Nontraumatic chronic subdural hemorrhage	I62.03
**35207817**	Nontraumatic extradural hemorrhage	I62.1
**35207818**	Nontraumatic intracranial hemorrhage, unspecified	I62.9
**1569193**	Cerebral infarction	I63
**1569194**	Cerebral infarction due to thrombosis of precerebral arteries	I63.0
**45601041**	Cerebral infarction due to thrombosis of unspecified precerebral artery	I63.00
**1569195**	Cerebral infarction due to thrombosis of vertebral artery	I63.01
**45552801**	Cerebral infarction due to thrombosis of basilar artery	I63.02
**1569196**	Cerebral infarction due to thrombosis of carotid artery	I63.03
**45533463**	Cerebral infarction due to thrombosis of other precerebral artery	I63.09
**1569197**	Cerebral infarction due to embolism of precerebral arteries	I63.1
**45567187**	Cerebral infarction due to embolism of unspecified precerebral artery	I63.10
**1569198**	Cerebral infarction due to embolism of vertebral artery	I63.11
**45581782**	Cerebral infarction due to embolism of basilar artery	I63.12
**1569199**	Cerebral infarction due to embolism of carotid artery	I63.13
**45605806**	Cerebral infarction due to embolism of other precerebral artery	I63.19
**1569200**	Cerebral infarction due to unspecified occlusion or stenosis of precerebral arteries	I63.2
**45552802**	Cerebral infarction due to unspecified occlusion or stenosis of unspecified precerebral arteries	I63.20
**1569201**	Cerebral infarction due to unspecified occlusion or stenosis of vertebral arteries	I63.21

© 2025 Zhang D et al. *JAMA Network Open*.

**45538396**	Cerebral infarction due to unspecified occlusion or stenosis of basilar artery	I63.22
**1569202**	Cerebral infarction due to unspecified occlusion or stenosis of carotid arteries	I63.23
**45548027**	Cerebral infarction due to unspecified occlusion or stenosis of other precerebral arteries	I63.29
**1569203**	Cerebral infarction due to thrombosis of cerebral arteries	I63.3
**45591474**	Cerebral infarction due to thrombosis of unspecified cerebral artery	I63.30
**1569204**	Cerebral infarction due to thrombosis of middle cerebral artery	I63.31
**1569205**	Cerebral infarction due to thrombosis of anterior cerebral artery	I63.32
**1569206**	Cerebral infarction due to thrombosis of posterior cerebral artery	I63.33
**1569207**	Cerebral infarction due to thrombosis of cerebellar artery	I63.34
**45576888**	Cerebral infarction due to thrombosis of other cerebral artery	I63.39
**1569208**	Cerebral infarction due to embolism of cerebral arteries	I63.4
**45552803**	Cerebral infarction due to embolism of unspecified cerebral artery	I63.40
**1569209**	Cerebral infarction due to embolism of middle cerebral artery	I63.41
**1569210**	Cerebral infarction due to embolism of anterior cerebral artery	I63.42
**1569211**	Cerebral infarction due to embolism of posterior cerebral artery	I63.43
**1569212**	Cerebral infarction due to embolism of cerebellar artery	I63.44
**45552805**	Cerebral infarction due to embolism of left cerebellar artery	I63.442
**37200509**	Cerebral infarction due to embolism of bilateral cerebellar arteries	I63.443
**45572099**	Cerebral infarction due to embolism of unspecified cerebellar artery	I63.449
**45601045**	Cerebral infarction due to embolism of other cerebral artery	I63.49
**1569213**	Cerebral infarction due to unspecified occlusion or stenosis of cerebral arteries	I63.5
**45552806**	Cerebral infarction due to unspecified occlusion or stenosis of unspecified cerebral artery	I63.50
**1569214**	Cerebral infarction due to unspecified occlusion or stenosis of middle cerebral artery	I63.51
**1569215**	Cerebral infarction due to unspecified occlusion or stenosis of anterior cerebral artery	I63.52
**1569216**	Cerebral infarction due to unspecified occlusion or stenosis of posterior cerebral artery	I63.53
**45601047**	Cerebral infarction due to unspecified occlusion or stenosis of other cerebral artery	I63.59
**35207819**	Cerebral infarction due to cerebral venous thrombosis, nonpyogenic	I63.6
**35207820**	Other cerebral infarction	I63.8
**1595597**	Other cerebral infarction due to occlusion or stenosis of small artery	I63.81
**1595598**	Other cerebral infarction	I63.89
**35207821**	Cerebral infarction, unspecified	I63.9
**35207829**	Cerebral aneurysm, nonruptured	I67.1
**35207830**	Cerebral atherosclerosis	I67.2
**35207833**	Moyamoya disease	I67.5
**35207835**	Cerebral arteritis, not elsewhere classified	I67.7

© 2025 Zhang D et al. *JAMA Network Open*.

**45533468**	Acute cerebrovascular insufficiency	I67.81
**45581787**	Cerebral ischemia	I67.82
**45562371**	Posterior reversible encephalopathy syndrome	I67.83
**45576890**	Other cerebrovascular disease	I67.89
**35207837**	Cerebrovascular disease, unspecified	I67.9
**35207838**	Cerebral amyloid angiopathy	I68.0
**35207839**	Cerebral arteritis in other diseases classified elsewhere	I68.2
**35207840**	Other cerebrovascular disorders in diseases classified elsewhere	I68.8
**45548036**	Unspecified sequelae of nontraumatic subarachnoid hemorrhage	I69.00
**45572104**	Cognitive deficits following nontraumatic subarachnoid hemorrhage	I69.01
**45586604**	Unspecified sequelae of nontraumatic intracerebral hemorrhage	I69.10
**45567195**	Cognitive deficits following nontraumatic intracerebral hemorrhage	I69.11
**45533472**	Unspecified sequelae of other nontraumatic intracranial hemorrhage	I69.20
**45562377**	Cognitive deficits following other nontraumatic intracranial hemorrhage	I69.21
**45605823**	Unspecified sequelae of other cerebrovascular disease	I69.80
**45567199**	Cognitive deficits following other cerebrovascular disease	I69.81
**45596225**	Unspecified sequelae of unspecified cerebrovascular disease	I69.90
**45548053**	Cognitive deficits following unspecified cerebrovascular disease	I69.91
**35207834**	Nonpyogenic thrombosis of intracranial venous system	I67.6
**35207682**	Other forms of angina pectoris	I20.8
**35207681**	Angina pectoris with documented spasm	I20.1
**35225418**	Presence of coronary angioplasty implant and graft	Z95.5
**35207702**	Old myocardial infarction	I25.2
**35207698**	Acute coronary thrombosis not resulting in myocardial infarction	I24.0
**45548013**	Coronary atherosclerosis due to lipid rich plaque	I25.83
**35207706**	Chronic ischemic heart disease, unspecified	I25.9
**45596199**	Chronic total occlusion of coronary artery	I25.82
**35207701**	Acute ischemic heart disease, unspecified	I24.9
**35207680**	Unstable angina	I20.0
**35225414**	Presence of aortocoronary bypass graft	Z95.1
**35207683**	Angina pectoris, unspecified	I20.9
**45567168**	Coronary atherosclerosis due to calcified coronary lesion	I25.84
**35207700**	Other forms of acute ischemic heart disease	I24.8
**35207705**	Silent myocardial ischemia	I25.6
**45605788**	Other forms of chronic ischemic heart disease	I25.89
**45585890**	Coronary angioplasty status	Z98.61
**45567183**	Other ill-defined heart diseases	I51.89
**45601038**	Unspecified diastolic (congestive) heart failure	I50.30

© 2025 Zhang D et al. *JAMA Network Open*.

**1326609**	Other heart failure	I50.89
**45576878**	Chronic systolic (congestive) heart failure	I50.22
**45543182**	Acute systolic (congestive) heart failure	I50.21
**45533456**	Chronic diastolic (congestive) heart failure	I50.32
**45591469**	Acute combined systolic (congestive) and diastolic (congestive) heart failure	I50.41
**1326607**	High output heart failure	I50.83
**35207793**	Heart failure, unspecified	I50.9
**45567181**	Acute on chronic combined systolic (congestive) and diastolic (congestive) heart failure	I50.43
**1326608**	End stage heart failure	I50.84
**45562355**	Acute on chronic diastolic (congestive) heart failure	I50.33
**35207792**	Left ventricular failure, unspecified	I50.1
**45567180**	Acute on chronic systolic (congestive) heart failure	I50.23
**45533457**	Unspecified combined systolic (congestive) and diastolic (congestive) heart failure	I50.40
**45605777**	Rheumatic heart failure	I09.81
**1326606**	Biventricular heart failure	I50.82
**45586587**	Unspecified systolic (congestive) heart failure	I50.20
**45586588**	Chronic combined systolic (congestive) and diastolic (congestive) heart failure	I50.42
**45548022**	Acute diastolic (congestive) heart failure	I50.31
**35207673**	Hypertensive heart and chronic kidney disease with heart failure and stage 1 through stage 4 chronic kidney disease, or unspecified chronic kidney disease	I13.0
**37200492**	Hypertensive crisis	I16
**1569124**	Secondary hypertension	I15
**35207670**	Hypertensive heart disease without heart failure	I11.9
**37200493**	Hypertensive urgency	I16.0
**35207676**	Hypertension secondary to other renal disorders	I15.1
**35207679**	Secondary hypertension, unspecified	I15.9
**45543164**	Hypertensive heart and chronic kidney disease without heart failure, with stage 1 through stage 4 chronic kidney disease, or unspecified chronic kidney disease	I13.10
**35207671**	Hypertensive chronic kidney disease with stage 5 chronic kidney disease or end stage renal disease	I12.0
**35207669**	Hypertensive heart disease with heart failure	I11.0
**35207674**	Hypertensive heart and chronic kidney disease with heart failure and with stage 5 chronic kidney disease, or end stage renal disease	I13.2
**35207672**	Hypertensive chronic kidney disease with stage 1 through stage 4 chronic kidney disease, or unspecified chronic kidney disease	I12.9
**35207677**	Hypertension secondary to endocrine disorders	I15.2
**1569122**	Hypertensive heart and chronic kidney disease	I13
**1569120**	Hypertensive heart disease	I11

© 2025 Zhang D et al. *JAMA Network Open*.

**45596188**	Hypertensive heart and chronic kidney disease without heart failure, with stage 5 chronic kidney disease, or end stage renal disease	I13.11
**37200495**	Hypertensive crisis, unspecified	I16.9
**1569123**	Hypertensive heart and chronic kidney disease without heart failure	I13.1
**35207678**	Other secondary hypertension	I15.8
**1569121**	Hypertensive chronic kidney disease	I12
**35207668**	Essential (primary) hypertension	I10
**35207675**	Renovascular hypertension	I15.0
**37200494**	Hypertensive emergency	I16.1
**45605875**	Postprocedural hypertension	I97.3
**45557538**	Coronary artery aneurysm	I25.41
**35207794**	Cardiac septal defect, acquired	I51.0
**35207799**	Myocardial degeneration	I51.5
**35207802**	Other heart disorders in diseases classified elsewhere	I52
**35207795**	Rupture of chordae tendineae, not elsewhere classified	I51.1
**35207801**	Heart disease, unspecified	I51.9
**35207800**	Cardiomegaly	I51.7
**35207796**	Rupture of papillary muscle, not elsewhere classified	I51.2
**45552874**	Unspecified disorder of circulatory system	I99.9
**35207877**	Hereditary hemorrhagic telangiectasia	I78.0
**35207876**	Disorder of arteries and arterioles, unspecified	I77.9
**35207872**	Celiac artery compression syndrome	I77.4
**35207879**	Other diseases of capillaries	I78.8
**35207878**	Nevus, non-neoplastic	I78.1
**45548097**	Other disorder of circulatory system	I99.8
**35207883**	Other disorders of arteries, arterioles and capillaries in diseases classified elsewhere	I79.8
**35207869**	Stricture of artery	I77.1
**45572134**	Other specified disorders of arteries and arterioles	I77.89
**35207870**	Rupture of artery	I77.2
**35207880**	Disease of capillaries, unspecified	I78.9
**35207882**	Aortitis in diseases classified elsewhere	I79.1
**35207873**	Necrosis of artery	I77.5
**35207868**	Arteriovenous fistula, acquired	I77.0
**35207871**	Arterial fibromuscular dysplasia	I77.3
**1326597**	Other secondary pulmonary hypertension	I27.29
**35207709**	Other secondary pulmonary hypertension	I27.2
**35207707**	Primary pulmonary hypertension	I27.0
**45601029**	Cor pulmonale (chronic)	I27.81
**1326598**	Eisenmenger's syndrome	I27.83

© 2025 Zhang D et al. *JAMA Network Open*.

**1326595**	Pulmonary hypertension due to lung diseases and hypoxia	I27.23
**35207708**	Kyphoscoliosis heart disease	I27.1
**35207713**	Other diseases of pulmonary vessels	I28.8
**1326593**	Secondary pulmonary arterial hypertension	I27.21
**1326594**	Pulmonary hypertension due to left heart disease	I27.22
**1326592**	Pulmonary hypertension, unspecified	I27.20
**35207711**	Arteriovenous fistula of pulmonary vessels	I28.0
**35207712**	Aneurysm of pulmonary artery	I28.1
**35207710**	Pulmonary heart disease, unspecified	I27.9
**45533442**	Other specified pulmonary heart diseases	I27.89
**1326596**	Chronic thromboembolic pulmonary hypertension	I27.24
**35207714**	Disease of pulmonary vessels, unspecified	I28.9

eTable 4. Medication List for GI Tract Symptoms or Disorders

**Name of medications**
Amitriptyline
Azithromycin
Bisacodyl
Cimetidine
Dicyclomine
Docusate
Docusate
Erythromycin
Esomeprazole
Famotidine
Hyoscyamine
Lactulose
Lansoprazole
Linaclotide
Linaclotide
Lubiprostone
Lubiprostone
Magnesium hydroxide
Metoclopramide
Nortriptyline
Omeprazole
Pantoprazole
Polyethylene glycol

© 2025 Zhang D et al. *JAMA Network Open*.

Ranitidine
Rifaximin
Senna
Senokot

eTable 5. List of Negative Control Outcomes

**Name of negative control outcomes**
Acne
Astigmatism
Autism or autistic disorder
Closed fracture of distal end of radius
Closed injury of head
Concussion
Contact dermatitis
Diaper rash
Displacements bone
Epilepsy
Falls
Foreign body in ear
Impetigo
Inguinal hernia
Injury of finger
Injury of free lower limb
Injury of head
Injury of left leg
Injury of right foot
Injury of right hand
Injury of right leg
Injury of upper extremity
Insect bite
Myopia
Obesity
Plagiocephaly
Scoliosis
Seizure
Snoring or obstructive sleep apnea
Speech delay
Speech dysfunction

© 2025 Zhang D et al. *JAMA Network Open*.

Sprain of ankle
Tinea capitis
Tinea corporis
Tongue tie
Umbilical hernia
Wax in ear or impacted cerumen

eTable 6. Adjusted Risk Ratio for the Subgroup Younger Than 5 Years

		Post acute		Chronic	
Category	Symptom	Est	95% CI	Est	95% CI
Signs or symptoms	Abdominal pain	1.151	(1.091, 1.215)	1.308	(1.262, 1.356)
	Bloating	1.319	(1.180, 1.474)	1.336	(1.210, 1.476)
	Constipation	1.252	(1.210, 1.296)	1.287	(1.252, 1.323)
	Diarrhea	1.421	(1.373, 1.472)	1.58	(1.533, 1.629)
	Nausea	1.478	(1.278,1.709)	1.345	(1.199, 1.510)
	Vomiting	1.357	(1.316,1.398)	1.463	(1.424,1.502)
Disorders	GERD	1.176	(1.113, 1.243)	1.288	(1.213, 1.368)
	IBS	0.739	(0.378,1.445)	1.15	(0.725,1.826)
	Functional dyspepsia	NA	NA	1.38	(1.081, 1.763)
Composite	Any signs or symptoms	1.318	(1.288,1.348)	1.361	(1.335,1.387)
	Any disorders	1.176	(1.113, 1.243)	1.281	(1.208, 1.359)
	Any visits related to GI	1.311	(1.282, 1.341)	1.358	(1.331, 1.385)

eTable 7. Adjusted Risk Ratio for the Subgroup Aged 5 to 11 Years

		Post acute		Chronic	
Category	Symptom	Est	95% CI	Est	95% CI
Signs or symptoms	Abdominal pain	1.249	(1.199, 1.301)	1.246	(1.206, 1.287)
	Bloating	1.339	(1.138,1.575)	1.321	(1.167, 1.496)
	Constipation	1.16	(1.109, 1.213)	1.178	(1.138,1.220)
	Diarrhea	1.324	(1.243, 1.410)	1.372	(1.305,1.444)
	Nausea	1.493	(1.374,1.621)	1.512	(1.419, 1.612)
	Vomiting	1.261	(1.201, 1.324)	1.322	(1.270, 1.376)
Disorders	GERD	1.211	(1.114, 1.316)	1.221	(1.148,1.299)
	IBS	0.98	(0.722, 1.330)	1.118	(0.908,1.375)
	Functional dyspepsia	NA	NA	1.298	(1.046,1.611)
Composite	Any signs or symptoms	1.234	(1.198,1.271)	1.224	(1.194, 1.254)
	Any disorders	1.211	(1.114, 1.316)	1.214	(1.145,1.287)
	Any visits related to GI	1.228	(1.192,1.265)	1.225	(1.196, 1.255)

eTable 8. Adjusted Risk Ratio for the Subgroup Aged 12 to 18 Years

		Post acute		Chronic	
Category	Symptom	Est	95% CI	Est	95% CI
Signs or symptoms	Abdominal pain	1.09	(1.046,1.137)	1.198	(1.160, 1.238)
	Bloating	1.129	(0.987, 1.291)	1.237	(1.123,1.363)
	Constipation	1.16	(1.100,1.222)	1.192	(1.143, 1.243)
	Diarrhea	1.243	(1.166,1.325)	1.285	(1.223, 1.351)
	Nausea	1.164	(1.097, 1.234)	1.297	(1.238, 1.359)
	Vomiting	1.215	(1.136, 1.299)	1.299	(1.232, 1.370)

© 2025 Zhang D et al. *JAMA Network Open*.

Disorders	GERD	1.117	(1.047, 1.193)	1.246	(1.185, 1.310)
	IBS	0.908	(0.797, 1.034)	1.056	(0.963,1.158)
	Functional dyspepsia	NA	NA	1.003	(0.867, 1.160)
Composite	Any signs or symptoms	1.158	(1.121, 1.196)	1.204	(1.173, 1.236)
	Any disorders	1.117	(1.047, 1.193)	1.192	(1.140, 1.247)
	Any visits related to GI	1.144	(1.108,1.181)	1.197	(1.167, 1.228)

eTable 9. Adjusted Risk Ratio for the Non-Hispanic Black Subgroup

		Post acute		Chronic	
Category	Symptom	Est	95% CI	Est	95% CI
Signs or symptoms	Abdominal pain	1.182	(1.106,1.264)	1.279	(1.217, 1.344)
	Bloating	1.163	(0.952, 1.420)	1.23	(1.041, 1.454)
	Constipation	1.273	(1.203, 1.347)	1.248	(1.193, 1.305)
	Diarrhea	1.39	(1.294, 1.492)	1.584	(1.493, 1.680)
	Nausea	1.287	(1.129, 1.467)	1.506	(1.361, 1.667)
	Vomiting	1.366	(1.288,1.448)	1.39	(1.324,1.460)
Disorders	GERD	1.238	(1.123,1.366)	1.343	(1.232, 1.464)
	IBS	NA	NA	0.987	(0.741, 1.315)
	Functional dyspepsia	NA	NA	1.522	(1.092, 2.121)
Composite	Any signs or symptoms	1.274	(1.225,1.325)	1.304	(1.263, 1.346)
	Any disorders	1.238	(1.123,1.366)	1.327	(1.222,1.440)
	Any visits related to GI	1.268	(1.220,1.318)	1.306	(1.265, 1.349)

eTable 10. Adjusted Risk Ratio for the Non-Hispanic White Subgroup

		Post acute		Chronic	
Category	Symptom	Est	95% CI	Est	95% CI
Signs or symptoms	Abdominal pain	1.141	(1.095, 1.188)	1.21	(1.174,1.248)
	Bloating	1.262	(1.123, 1.418)	1.243	(1.135,1.361)
	Constipation	1.173	(1.127, 1.222)	1.146	(1.111, 1.183)
	Diarrhea	1.401	(1.338,1.467)	1.417	(1.364, 1.472)
	Nausea	1.292	(1.210, 1.379)	1.362	(1.293, 1.434)
	Vomiting	1.351	(1.296, 1.409)	1.347	(1.300, 1.395)
Disorders	GERD	1.17	(1.109, 1.234)	1.288	(1.230,1.348)
	IBS	0.903	(0.780, 1.045)	1.082	(0.974,1.202)
	Functional dyspepsia	NA	NA	1.051	(0.902, 1.225)
Composite	Any signs or symptoms	1.263	(1.231, 1.297)	1.235	(1.209, 1.262)
	Any disorders	1.17	(1.109, 1.234)	1.25	(1.198,1.304)
	Any visits related to GI	1.254	(1.222, 1.286)	1.232	(1.207, 1.259)

eTable 11. Adjusted Risk Ratio for the Hispanic Subgroup

		Post acute		Chronic	
Category	Symptom	Est	95% CI	Est	95% CI
Signs or symptoms	Abdominal pain	1.134	(1.083,1.188)	1.254	(1.210, 1.299)
	Bloating	1.342	(1.165,1.546)	1.47	(1.312,1.648)
	Constipation	1.209	(1.156,1.265)	1.271	(1.227, 1.317)
	Diarrhea	1.423	(1.357, 1.493)	1.501	(1.441, 1.563)
	Nausea	1.251	(1.146, 1.367)	1.414	(1.319, 1.516)
	Vomiting	1.286	(1.234,1.341)	1.443	(1.393, 1.496)
Disorders	GERD	1.221	(1.123,1.327)	1.328	(1.238, 1.425)
	IBS	0.873	(0.651, 1.170)	1.245	(1.024, 1.515)
	Functional dyspepsia	NA	NA	1.389	(1.115,1.730)
Composite	Any signs or symptoms	1.256	(1.221,1.293)	1.318	(1.287, 1.351)

© 2025 Zhang D et al. *JAMA Network Open*.

	Any disorders	1.221	(1.123, 1.327)	1.324	(1.240, 1.414)
	Any visits related to GI	1.251	(1.216,1.288)	1.317	(1.286,1.349)

© 2025 Zhang D et al. *JAMA Network Open*.

eTable 12. Adjusted Risk Ratio for the Asian American and Pacific Islander Subgroup

		Post acute		Chronic	
Category	Symptom	Est	95% CI	Est	95% CI
Signs or symptoms	Abdominal pain	1.185	(1.040, 1.350)	1.215	(1.100,1.341)
	Bloating	1.17	(0.804, 1.702)	1.12	(0.821, 1.526)
	Constipation	1.144	(1.020, 1.283)	1.263	(1.151, 1.384)
	Diarrhea	1.282	(1.115,1.474)	1.492	(1.327, 1.677)
	Nausea	1.159	(0.889,1.511)	1.029	(0.835,1.268)
	Vomiting	1.364	(1.225,1.520)	1.361	(1.237, 1.496)
Disorders	GERD	1.077	(0.882, 1.314)	1.105	(0.918,1.330)
	IBS	NA	NA	NA	NA
	Functional dyspepsia	NA	NA	0.878	(0.483, 1.596)
Composite	Any signs or symptoms	1.279	(1.186,1.380)	1.269	(1.191, 1.353)
	Any disorders	1.077	(0.882, 1.314)	1.06	(0.889, 1.263)
	Any visits related to GI	1.261	(1.170, 1.359)	1.27	(1.192, 1.354)

eTable 13. Adjusted Risk Ratio for the Subgroup With Obesity

		Post acute		Chronic	
Category	Symptom	Est	95% CI	Est	95% CI
Signs or symptoms	Abdominal pain	1.154	(1.110, 1.201)	1.223	(1.187, 1.260)
	Bloating	1.2	(1.046, 1.376)	1.258	(1.138,1.390)
	Constipation	1.152	(1.105,1.201)	1.201	(1.165,1.239)
	Diarrhea	1.303	(1.238, 1.371)	1.376	(1.321, 1.433)
	Nausea	1.204	(1.123, 1.290)	1.361	(1.291, 1.435)
	Vomiting	1.303	(1.245, 1.365)	1.332	(1.284, 1.381)
Disorders	GERD	1.177	(1.100,1.260)	1.233	(1.172, 1.297)
	IBS	0.921	(0.765,1.110)	1.084	(0.957, 1.227)
	Functional dyspepsia	NA	NA	1.218	(1.032, 1.438)
Composite	Any signs or symptoms	1.204	(1.171, 1.237)	1.223	(1.196,1.249)
	Any disorders	1.177	(1.100,1.260)	1.213	(1.157, 1.272)
	Any visits related to GI	1.189	(1.157, 1.222)	1.216	(1.190, 1.242)

eTable 14. Adjusted Risk Ratio for the Subgroup Without Obesity

		Post acute		Chronic	
Category	Symptom	Est	95% CI	Est	95% CI
Signs or symptoms	Abdominal pain	1.141	(1.100,1.184)	1.244	(1.210,1.279)
	Bloating	1.278	(1.161, 1.408)	1.316	(1.215,1.426)
	Constipation	1.241	(1.202, 1.282)	1.247	(1.215,1.280)
	Diarrhea	1.413	(1.363, 1.464)	1.51	(1.465, 1.556)
	Nausea	1.295	(1.212, 1.383)	1.355	(1.285,1.428)
	Vomiting	1.344	(1.303, 1.386)	1.445	(1.407, 1.484)
Disorders	GERD	1.187	(1.130, 1.246)	1.283	(1.225,1.343)
	IBS	0.866	(0.736, 1.019)	1.049	(0.933, 1.180)
	Functional dyspepsia	NA	NA	1.094	(0.942, 1.271)
Composite	Any signs or symptoms	1.285	(1.258,1.313)	1.315	(1.291, 1.338)
	Any disorders	1.187	(1.130, 1.246)	1.249	(1.197, 1.304)
	Any visits related to GI	1.285	(1.258,1.313)	1.312	(1.289, 1.336)

© 2025 Zhang D et al. *JAMA Network Open*.

eTable 15. Adjusted Risk Ratio for the Female Subgroup

		Post acute		Chronic	
Category	Symptom	Est	95% CI	Est	95% CI
Signs or symptoms	Abdominal pain	1.117	(1.080, 1.156)	1.217	(1.186,1.248)
	Bloating	1.257	(1.134, 1.393)	1.287	(1.188,1.394)
	Constipation	1.176	(1.138, 1.215)	1.21	(1.179,1.241)
	Diarrhea	1.379	(1.324, 1.436)	1.478	(1.429, 1.529)
	Nausea	1.225	(1.157, 1.297)	1.357	(1.299, 1.419)
	Vomiting	1.298	(1.254, 1.344)	1.373	(1.333,1.414)
Disorders	GERD	1.178	(1.116, 1.243)	1.311	(1.253,1.371)
	IBS	0.902	(0.782, 1.040)	1.031	(0.932,1.141)
	Functional dyspepsia	NA	NA	1.152	(1.008,1.316)
Composite	Any signs or symptoms	1.224	(1.198,1.252)	1.26	(1.238,1.283)
	Any disorders	1.178	(1.116,1.243)	1.262	(1.211, 1.315)
	Any visits related to GI	1.223	(1.196, 1.250)	1.267	(1.244, 1.289)

eTable 16. Adjusted Risk Ratio for the Male Subgroup

		Post acute		Chronic	
Category	Symptom	Est	95% CI	Est	95% CI
Signs or symptoms	Abdominal pain	1.175	(1.130, 1.222)	1.274	(1.237, 1.313)
	Bloating	1.298	(1.161, 1.450)	1.307	(1.192,1.434)
	Constipation	1.235	(1.192, 1.280)	1.252	(1.217, 1.288)
	Diarrhea	1.403	(1.352, 1.456)	1.479	(1.433, 1.526)
	Nausea	1.338	(1.242, 1.442)	1.414	(1.330,1.503)
	Vomiting	1.36	(1.316, 1.406)	1.415	(1.375,1.456)
Disorders	GERD	1.211	(1.148,1.277)	1.253	(1.194,1.314)
	IBS	0.913	(0.743,1.122)	1.191	(1.032, 1.376)
	Functional dyspepsia	NA	NA	1.089	(0.905, 1.309)
Composite	Any signs or symptoms	1.297	(1.268, 1.327)	1.3	(1.275,1.325)
	Any disorders	1.211	(1.148,1.277)	1.246	(1.191, 1.303)
	Any visits related to GI	1.288	(1.260, 1.317)	1.292	(1.267, 1.316)

eTable 17. Adjusted Risk Ratio for the Subgroup With a Medical History of Diabetes

		Post acute		Chronic	
Category	Symptom	Est	95% CI	Est	95% CI
Signs or symptoms	Abdominal pain	1.177	(0.917, 1.509)	1.313	(1.099,1.568)
	Bloating	NA	NA	1.815	(1.097, 3.003)
	Constipation	1.1	(0.816,1.484)	1.218	(0.963, 1.541)
	Diarrhea	1.556	(1.157, 2.092)	NA	NA
	Nausea	1.074	(0.737, 1.566)	1.537	(1.186,1.991)
	Vomiting	1.594	(1.212, 2.097)	1.486	(1.190, 1.855)
Disorders	GERD	NA	NA	1.403	(1.053,1.869)
	IBS	NA	NA	NA	NA
	Functional dyspepsia	NA	NA	NA	NA
Composite	Any signs or symptoms	1.295	(1.069, 1.569)	1.354	(1.162,1.578)
	Any disorders	NA	NA	1.427	(1.091,1.867)
	Any visits related to GI	1.248	(1.026,1.518)	1.312	(1.126,1.529)

eTable 18. Adjusted Risk Ratio for the Subgroup Without a Medical History of Diabetes

		Post acute		Chronic	
Category	Symptom	Est	95% CI	Est	95% CI

© 2025 Zhang D et al. *JAMA Network Open*.

Signs or symptoms	Abdominal pain	1.141	(1.112,1.170)	1.24	(1.216,1.264)
	Bloating	1.279	(1.186,1.380)	1.284	(1.209, 1.365)
	Constipation	1.203	(1.174, 1.232)	1.229	(1.206,1.253)
	Diarrhea	1.396	(1.358,1.435)	1.48	(1.446, 1.515)
	Nausea	1.273	(1.216,1.332)	1.374	(1.326, 1.425)
	Vomiting	1.33	(1.298,1.362)	1.395	(1.366, 1.424)
Disorders	GERD	1.191	(1.147, 1.237)	1.274	(1.233,1.316)
	IBS	0.888	(0.789, 0.999)	1.068	(0.982, 1.160)
	Functional dyspepsia	NA	NA	1.122	(1.006,1.251)
Composite	Any signs or symptoms	1.26	(1.241, 1.281)	1.28	(1.264, 1.297)
	Any disorders	1.191	(1.147, 1.237)	1.244	(1.206,1.283)
	Any visits related to GI	1.254	(1.235,1.274)	1.278	(1.261, 1.295)

eTable 19. Adjusted Risk Ratio for the Subgroup With a Medical History of Cardiovascular Diseases

		Post acute		Chronic	
Category	Symptom	Est	95% CI	Est	95% CI
Signs or symptoms	Abdominal pain	1.352	(1.176, 1.555)	1.56	(1.406,1.732)
	Bloating	1.397	(1.128,1.729)	1.357	(1.129,1.631)
	Constipation	1.39	(1.245, 1.553)	1.332	(1.215,1.461)
	Diarrhea	1.555	(1.373,1.762)	1.67	(1.504,1.855)
	Nausea	1.29	(1.061, 1.567)	1.73	(1.465, 2.044)
	Vomiting	1.377	(1.227, 1.546)	1.491	(1.353,1.643)
Disorders	GERD	1.295	(1.126,1.490)	1.524	(1.353,1.717)
	IBS	NA	NA	1.721	(1.131, 2.619)
	Functional dyspepsia	NA	NA	1.233	(0.864,1.760)
Composite	Any signs or symptoms	1.445	(1.318,1.584)	1.37	(1.266,1.482)
	Any disorders	1.295	(1.126,1.490)	1.556	(1.385,1.747)
	Any visits related to GI	1.375	(1.248, 1.513)	1.319	(1.214,1.433)

eTable 20. Adjusted Risk Ratio for the Subgroup Without a Medical History of Cardiovascular Diseases

		Post acute		Chronic	
Category	Symptom	Est	95% CI	Est	95% CI
Signs or symptoms	Abdominal pain	1.134	(1.105,1.164)	1.23	(1.206, 1.255)
	Bloating	1.239	(1.143,1.344)	1.279	(1.199, 1.364)
	Constipation	1.195	(1.166,1.225)	1.225	(1.202, 1.250)
	Diarrhea	1.386	(1.347, 1.425)	1.469	(1.434, 1.504)
	Nausea	1.27	(1.212, 1.331)	1.365	(1.316, 1.415)
	Vomiting	1.33	(1.298,1.363)	1.39	(1.361, 1.419)
Disorders	GERD	1.191	(1.146,1.239)	1.262	(1.219, 1.305)
	IBS	0.873	(0.774, 0.985)	1.052	(0.967, 1.144)
	Functional dyspepsia	NA	NA	1.122	(1.002,1.257)
Composite	Any signs or symptoms	1.256	(1.236,1.276)	1.277	(1.261, 1.294)
	Any disorders	1.191	(1.146,1.239)	1.229	(1.191, 1.268)
	Any visits related to GI	1.251	(1.232, 1.271)	1.276	(1.259,1.293)

eTable 21. Adjusted Risk Ratio for the Subgroup Without Hospitalization

		Post acute		Chronic	
Category	Symptom	Est	95% CI	Est	95% CI
Signs or symptoms	Abdominal pain	1.127	(1.097, 1.158)	1.222	(1.198,1.247)
	Bloating	1.282	(1.176,1.397)	1.281	(1.197, 1.370)
	Constipation	1.198	(1.167, 1.229)	1.213	(1.188,1.237)
	Diarrhea	1.362	(1.323,1.402)	1.446	(1.411, 1.482)

© 2025 Zhang D et al. *JAMA Network Open*.

	Nausea	1.268	(1.207, 1.333)	1.354	(1.304, 1.407)
	Vomiting	1.309	(1.275,1.342)	1.373	(1.344, 1.403)
Disorders	GERD	1.243	(1.192, 1.297)	1.282	(1.237, 1.328)
	IBS	0.934	(0.823,1.060)	1.105	(1.012, 1.207)
	Functional dyspepsia	NA	NA	1.103	(0.979, 1.242)
Composite	Any signs or symptoms	1.245	(1.224,1.265)	1.261	(1.244, 1.278)
	Any disorders	1.243	(1.192, 1.297)	1.26	(1.219,1.302)
	Any visits related to GI	1.244	(1.224,1.265)	1.262	(1.245,1.279)

eTable 22. Adjusted Risk Ratio for the Subgroup With Hospitalization

		Post acute		Chronic	
Category	Symptom	Est	95% CI	Est	95% CI
Signs or symptoms	Abdominal pain	1.413	(1.281, 1.558)	1.536	(1.416,1.666)
	Bloating	NA	NA	1.647	(1.404,1.931)
	Constipation	1.531	(1.412, 1.659)	1.556	(1.448,1.671)
	Diarrhea	1.837	(1.668, 2.024)	1.952	(1.794, 2.124)
	Nausea	1.613	(1.414,1.841)	1.923	(1.701, 2.173)
	Vomiting	1.635	(1.507, 1.774)	1.842	(1.710,1.983)
Disorders	GERD	1.58	(1.433, 1.742)	1.787	(1.621,1.969)
	IBS	1.096	(0.755,1.592)	1.343	(1.016,1.774)
	Functional dyspepsia	NA	NA	NA	NA
Composite	Any signs or symptoms	1.605	(1.511, 1.704)	1.642	(1.554,1.735)
	Any disorders	1.58	(1.433, 1.742)	1.709	(1.557, 1.876)
	Any visits related to GI	1.607	(1.515,1.705)	1.633	(1.544, 1.726)

eTable 23. Adjusted Risk Ratio for the Subgroup With ICU Admission

		Post acute		Chronic	
Category	Symptom	Est	95% CI	Est	95% CI
Signs or symptoms	Abdominal pain	2.025	(1.577, 2.600)	2.57	(2.105, 3.137)
	Bloating	2.551	(1.832, 3.552)	NA	NA
	Constipation	2.107	(1.785, 2.487)	2.057	(1.766, 2.397)
	Diarrhea	2.183	(1.746,2.730)	2.787	(2.320, 3.347)
	Nausea	2.735	(1.978,3.782)	3.713	(2.703, 5.102)
	Vomiting	1.97	(1.658, 2.342)	2.279	(1.938,2.680)
Disorders	GERD	1.335	(1.103, 1.615)	2.04	(1.701, 2.448)
	IBS	NA	NA	NA	NA
	Functional dyspepsia	NA	NA	NA	NA
Composite	Any signs or symptoms	1.978	(1.730, 2.262)	2.089	(1.837, 2.376)
	Any disorders	1.335	(1.103, 1.615)	2.073	(1.732, 2.481)
	Any visits related to GI	1.793	(1.574, 2.042)	2.013	(1.768,2.292)

eTable 24. Adjusted Risk Ratio for the Asymptomatic Subgroup

		Post acute		Chronic	
Category	Symptom	Est	95% CI	Est	95% CI
Signs or symptoms	Abdominal pain	1.231	(1.187, 1.277)	1.294	(1.261, 1.329)
	Bloating	1.279	(1.142, 1.432)	1.331	(1.221, 1.450)
	Constipation	1.225	(1.185,1.268)	1.241	(1.210, 1.274)
	Diarrhea	1.437	(1.379, 1.497)	1.485	(1.437, 1.535)
	Nausea	1.334	(1.245, 1.430)	1.449	(1.378,1.523)
	Vomiting	1.438	(1.387, 1.491)	1.435	(1.393, 1.478)
Disorders	GERD	1.191	(1.128,1.259)	1.283	(1.227, 1.341)
	IBS	1.201	(1.010, 1.429)	1.184	(1.054, 1.331)

© 2025 Zhang D et al. *JAMA Network Open*.

	Functional dyspepsia	NA	NA	1.106	(0.948,1.290)
Composite	Any signs or symptoms	1.317	(1.288,1.347)	1.299	(1.277, 1.322)
	Any disorders	1.191	(1.128,1.259)	1.271	(1.219, 1.326)
	Any visits related to GI	1.304	(1.275,1.333)	1.295	(1.272, 1.318)

eTable 25. Adjusted Risk Ratio for the Mild Subgroup

		Post acute		Chronic	
Category	Symptom	Est	95% CI	Est	95% CI
Signs or symptoms	Abdominal pain	0.983	(0.942, 1.025)	1.091	(1.056,1.128)
	Bloating	1.206	(1.050, 1.386)	1.162	(1.036,1.303)
	Constipation	1.103	(1.057, 1.150)	1.139	(1.101, 1.179)
	Diarrhea	1.238	(1.183,1.295)	1.358	(1.307, 1.412)
	Nausea	1.1	(1.021,1.184)	1.199	(1.129, 1.274)
	Vomiting	1.176	(1.130,1.223)	1.248	(1.206, 1.291)
Disorders	GERD	1.172	(1.095,1.256)	1.184	(1.113, 1.260)
	IBS	0.755	(0.617, 0.924)	1.009	(0.875,1.163)
	Functional dyspepsia	NA	NA	1.071	(0.883,1.298)
Composite	Any signs or symptoms	1.127	(1.098, 1.157)	1.16	(1.134, 1.187)
	Any disorders	1.172	(1.095, 1.256)	1.147	(1.083, 1.215)
	Any visits related to GI	1.129	(1.100,1.160)	1.16	(1.134, 1.187)

eTable 26. Adjusted Risk Ratio for the Moderate Subgroup

		Post acute		Chronic	
Category	Symptom	Est	95% CI	Est	95% CI
Signs or symptoms	Abdominal pain	1.28	(1.161, 1.411)	1.374	(1.268,1.489)
	Bloating	NA	NA	1.331	(1.092, 1.622)
	Constipation	1.387	(1.268,1.517)	1.41	(1.307, 1.522)
	Diarrhea	1.591	(1.450,1.744)	1.743	(1.606,1.893)
	Nausea	1.703	(1.467, 1.975)	1.849	(1.621, 2.109)
	Vomiting	1.429	(1.321, 1.546)	1.64	(1.526,1.762)
Disorders	GERD	1.468	(1.297, 1.662)	1.731	(1.543, 1.942)
	IBS	NA	NA	0.943	(0.681, 1.306)
	Functional dyspepsia	NA	NA	NA	NA
Composite	Any signs or symptoms	1.362	(1.282, 1.448)	1.52	(1.441, 1.604)
	Any disorders	1.468	(1.297, 1.662)	1.633	(1.463, 1.823)
	Any visits related to GI	1.383	(1.301, 1.470)	1.521	(1.440,1.607)

eTable 27. Adjusted Risk Ratio for the Severe Subgroup

		Post acute		Chronic	
Category	Symptom	Est	95% CI	Est	95% CI
Signs or symptoms	Abdominal pain	1.406	(1.199,1.649)	1.586	(1.400,1.798)
	Bloating	1.357	(1.071,1.718)	1.887	(1.538,2.316)
	Constipation	1.611	(1.436,1.806)	1.626	(1.471, 1.797)
	Diarrhea	1.697	(1.475,1.953)	1.917	(1.704, 2.158)
	Nausea	1.681	(1.355, 2.086)	NA	NA
	Vomiting	1.556	(1.391, 1.739)	1.823	(1.647,2.018)
Disorders	GERD	1.561	(1.375,1.771)	2.025	(1.789, 2.293)
	IBS	NA	NA	NA	NA
	Functional dyspepsia	NA	NA	NA	NA
Composite	Any signs or symptoms	1.561	(1.431, 1.703)	1.651	(1.526,1.787)
	Any disorders	1.561	(1.375,1.771)	2.06	(1.823, 2.327)
	Any visits related to GI	1.534	(1.409,1.670)	1.652	(1.525,1.791)

© 2025 Zhang D et al. *JAMA Network Open*.

eTable 28. Adjusted Risk Ratio for the Pre-Delta Subgroup

		Post acute		Chronic	
Category	Symptom	Est	95% CI	Est	95% CI
Signs or symptoms	Abdominal pain	1.196	(1.136,1.260)	1.293	(1.251, 1.336)
	Bloating	1.221	(1.052, 1.416)	1.367	(1.235,1.512)
	Constipation	1.238	(1.178,1.301)	1.315	(1.271,1.360)
	Diarrhea	1.471	(1.383, 1.563)	1.65	(1.584,1.719)
	Nausea	1.253	(1.150, 1.366)	1.389	(1.310, 1.472)
	Vomiting	1.322	(1.249, 1.400)	1.551	(1.494, 1.610)
Disorders	GERD	1.2	(1.116, 1.291)	1.297	(1.227, 1.371)
	IBS	0.811	(0.664, 0.990)	1.117	(0.983,1.268)
	Functional dyspepsia	NA	NA	1.335	(1.125,1.584)
Composite	Any signs or symptoms	1.294	(1.251, 1.338)	1.359	(1.328,1.391)
	Any disorders	1.2	(1.116,1.291)	1.278	(1.215,1.345)
	Any visits related to GI	1.28	(1.238,1.323)	1.347	(1.316,1.379)

eTable 29. Adjusted Risk Ratio for the Delta Subgroup

		Post acute		Chronic	
Category	Symptom	Est	95% CI	Est	95% CI
Signs or symptoms	Abdominal pain	1.213	(1.154, 1.275)	1.218	(1.177, 1.260)
	Bloating	1.272	(1.075,1.504)	1.291	(1.150,1.448)
	Constipation	1.182	(1.124,1.242)	1.154	(1.114, 1.196)
	Diarrhea	1.387	(1.312, 1.467)	1.446	(1.386,1.508)
	Nausea	1.358	(1.239, 1.488)	1.317	(1.234, 1.406)
	Vomiting	1.36	(1.296, 1.427)	1.362	(1.314, 1.413)
Disorders	GERD	1.182	(1.090, 1.282)	1.24	(1.166, 1.319)
	IBS	0.813	(0.634,1.044)	0.992	(0.847, 1.162)
	Functional dyspepsia	NA	NA	1.163	(0.945,1.433)
Composite	Any signs or symptoms	1.281	(1.242, 1.322)	1.235	(1.207, 1.265)
	Any disorders	1.182	(1.090, 1.282)	1.206	(1.138,1.277)
	Any visits related to GI	1.269	(1.230,1.310)	1.231	(1.202,1.260)

eTable 30. Adjusted Risk Ratio for the Omicron Subgroup

		Post acute		Chronic	
Category	Symptom	Est	95% CI	Est	95% CI
Signs or symptoms	Abdominal pain	1.111	(1.071, 1.153)	1.176	(1.137, 1.216)
	Bloating	1.315	(1.183,1.461)	1.266	(1.141, 1.404)
	Constipation	1.208	(1.168,1.250)	1.217	(1.179, 1.257)
	Diarrhea	1.386	(1.334,1.440)	1.387	(1.335,1.441)
	Nausea	1.298	(1.214, 1.388)	1.41	(1.322, 1.504)
	Vomiting	1.343	(1.300, 1.388)	1.307	(1.264, 1.351)
Disorders	GERD	1.193	(1.131, 1.259)	1.271	(1.203, 1.344)
	IBS	1.076	(0.893, 1.297)	1.143	(0.979, 1.333)
	Functional dyspepsia	NA	NA	0.94	(0.777, 1.136)
Composite	Any signs or symptoms	1.259	(1.231, 1.287)	1.24	(1.213,1.267)
	Any disorders	1.193	(1.131, 1.259)	1.235	(1.172,1.301)
	Any visits related to GI	1.255	(1.228,1.283)	1.24	(1.213,1.267)

eTable 31. Calibrated Risk Ratios for the GI Tract Outcomes During the Postacute or Chronic Phase

		Post acute		Chronic	
Category	Symptom	Est	95% CI	Est	95% CI

© 2025 Zhang D et al. *JAMA Network Open*.

Signs or symptoms	Abdominal pain	1.073	(0.822, 1.402)	0.98	(0.706,1.361)
	Bloating	1.207	(0.902, 1.616)	0.997	(0.701, 1.416)
	Constipation	1.163	(0.852, 1.587)	0.953	(0.635,1.429)
	Diarrhea	1.311	(0.994, 1.730)	1.161	(0.817, 1.652)
	Nausea	1.187	(0.910,1.547)	1.06	(0.748,1.502)
	Vomiting	1.256	(0.943, 1.673)	1.102	(0.758,1.602)
Disorders	GERD	1.14	(0.850, 1.530)	1.001	(0.686,1.459)
	IBS	NA	NA	0.844	(0.606,1.175)
	Functional dyspepsia	NA	NA	0.87	(0.612, 1.237)
Composite	Any signs or symptoms	1.221	(0.885, 1.685)	1.032	(0.696,1.529)
	Any disorders	1.14	(0.850, 1.530)	0.97	(0.665,1.414)
	Any visits related to GI	1.219	(0.879, 1.691)	1.057	(0.705,1.586)

eTable 32. Incidence by Person-Years for Chronic Phase by Documented COVID-19 Infection Status

Category	Outcome	Chronic phase Person-years/Total Person-years (%)	
		COVID-19 positive	COVID-19 negative
Signs and symptoms	Abdominal Pain	21749.43/420563.36 (5.17%)	47713.75/1229232.43 (3.88%)
	Bloating	2161.39/446028.77 (0.48%)	5012.35/1292646.68 (0.39%)
	Constipation	21102.90/405982.28 (5.20%)	50700.69/1188937.29 (4.26%)
	Diarrhea	15540.27/427664.53 (3.63%)	29646.44/1251210.66 (2.37%)
	Nausea	6737.38/443318.04 (1.52%)	12721.85/1287901.61 (0.99%)
	Vomiting	19234.81/416644.35 (4.62%)	41116.65/1217958.37 (3.38%)
Disorders	GERD	7416.91/423151.66 (1.75%)	16842.03/1235608.67 (1.36%)
	IBS	1155.74/451225.58 (0.26%)	2949.47/1305485.61 (0.23%)
	Functional dyspepsia	689.07/451642.72 (0.15%)	1627.22/1306643.71 (0.12%)
Composite	Any signs or symptoms	45989.03/346295.00 (13.28%)	105251.89/1038523.43 (10.13%)
	Any disorders	8468.83/421546.39 (2.01%)	19546.86/1231320.26 (1.59%)
	Any visits related to GI	45788.84/333704.02 (13.72%)	105598.05/ 1005378.31 (10.50%)

Abbreviation: GERD, gastroesophageal reflux disease; IBS, irritable bowel syndrome; GI, gastrointestinal.

[a]reported raw numbers and incidence for COVID-19 positive cohort (with documented COVID-19 infection) and COVID-19 negative cohort (without documented COVID-19 infection). Chronic phase is from 180 days to 729 days after the cohort entry date.

[b]Incidence is defined as the person-years of patients who developed specified GI symptoms or disorders during the post-acute or chronic phase, divided by the total person-years for the at-risk population (those without these corresponding outcomes at the baseline period).

eTable 33. Adjusted Risk Ratios Adding GI-Related Visits During the Acute Phase to Propensity Score

		Post acute		Chronic	
Category	Symptom	Est	95% CI	Est	95% CI
Signs or symptoms	Abdominal pain	1.155	[1.121, 1.190]	1.240	[1.212, 1.269]
	Bloating	1.427	[1.278, 1.592]	1.381	[1.264, 1.510]

© 2025 Zhang D et al. *JAMA Network Open*.

	Constipation	1.240	[1.205, 1.276]	1.255	[1.228, 1.284]
	Diarrhea	1.424	[1.376, 1.474]	1.500	[1.457, 1.543]
	Nausea	1.392	[1.313, 1.474]	1.459	[1.395, 1.527]
	Vomiting	1.374	[1.335, 1.414]	1.408	[1.374, 1.443]
Disorders	GERD	1.280	[1.222, 1.341]	1.314	[1.261, 1.369]
	IBS	1.395	[1.146, 1.697]	1.411	[1.247, 1.596]
	Functional dyspepsia	1.446	[1.160, 1.802]	1.199	[1.017, 1.414]
Composite	Any signs or symptoms	1.282	[1.262, 1.303]	1.291	[1.274, 1.308]
	Any disorders	1.280	[1.222, 1.341]	1.312	[1.263, 1.364]
	Any visits related to GI	1.273	[1.253, 1.293]	1.286	[1.269, 1.303]

eTable 34. Adjusted Risk Ratios Adding GI-Related Medications Before Postacute Phase to Propensity Score

		Post acute		Chronic	
Category	Symptom	Est	95% CI	Est	95% CI
Signs or symptoms	Abdominal pain	1.158	[1.123, 1.193]	1.244	[1.215, 1.272]
	Bloating	1.443	[1.293, 1.611]	1.393	[1.274, 1.522]
	Constipation	1.262	[1.226, 1.299]	1.271	[1.243, 1.300]
	Diarrhea	1.416	[1.369, 1.466]	1.498	[1.455, 1.541]
	Nausea	1.410	[1.330, 1.494]	1.466	[1.402, 1.534]
	Vomiting	1.371	[1.332, 1.410]	1.408	[1.374, 1.443]
Disorders	GERD	1.311	[1.251, 1.373]	1.341	[1.287, 1.397]
	IBS	1.425	[1.171, 1.735]	1.423	[1.257, 1.610]
	Functional dyspepsia	1.465	[1.175, 1.827]	1.234	[1.046, 1.455]
Composite	Any signs or symptoms	1.287	[1.266, 1.308]	1.296	[1.279, 1.314]
	Any disorders	1.311	[1.251, 1.373]	1.338	[1.287, 1.391]
	Any visits related to GI	1.280	[1.260, 1.300]	1.293	[1.276, 1.310]

eTable 35. Adjusted Risk Ratios for the Test Positivity Subgroup

		Post acute		Chronic	
Category	Symptom	Est	95% CI	Est	95% CI
Signs or symptoms	Abdominal pain	1.089	[1.038, 1.143]	1.180	[1.139, 1.222]
	Bloating	1.167	[0.967, 1.407]	1.279	[1.115, 1.467]
	Constipation	1.088	[1.037, 1.141]	1.156	[1.116, 1.198]
	Diarrhea	1.284	[1.213, 1.358]	1.421	[1.359, 1.486]
	Nausea	1.185	[1.081, 1.299]	1.406	[1.316, 1.502]
	Vomiting	1.283	[1.224, 1.344]	1.262	[1.214, 1.311]
Disorders	GERD	0.992	[0.912, 1.078]	1.115	[1.042, 1.194]
	IBS	1.032	[0.740, 1.440]	1.170	[0.965, 1.419]
	Functional dyspepsia	1.135	[0.779, 1.655]	0.948	[0.727, 1.236]
Composite	Any signs or symptoms	1.170	[1.140, 1.202]	1.211	[1.186, 1.236]
	Any disorders	0.992	[0.912, 1.078]	1.098	[1.030, 1.170]
	Any visits related to GI	1.154	[1.125, 1.184]	1.200	[1.176, 1.225]

eTable 36. Adjusted Risk Ratios for the COVID-19 Diagnosis Subgroup

		Post acute		Chronic	
Category	Symptom	Est	95% CI	Est	95% CI
Signs or symptoms	Abdominal pain	1.164	[1.124, 1.205]	1.277	[1.244, 1.312]
	Bloating	1.494	[1.320, 1.691]	1.402	[1.264, 1.555]
	Constipation	1.28	[1.239, 1.322]	1.307	[1.274, 1.341]
	Diarrhea	1.475	[1.419, 1.533]	1.544	[1.494, 1.596]
	Nausea	1.46	[1.367, 1.559]	1.480	[1.404, 1.560]

© 2025 Zhang D et al. *JAMA Network Open*.

	Vomiting	1.411	[1.366, 1.457]	1.479	[1.438, 1.521]
Disorders	GERD	1.347	[1.279, 1.418]	1.397	[1.334, 1.464]
	IBS	1.479	[1.186, 1.845]	1.530	[1.327, 1.764]
	Functional dyspepsia	1.412	[1.099, 1.815]	1.299	[1.078, 1.567]
Composite	Any signs or symptoms	1.314	[1.290, 1.339]	1.333	[1.312, 1.354]
	Any disorders	1.347	[1.279, 1.418]	1.402	[1.343, 1.465]
	Any visits related to GI	1.306	[1.283, 1.329]	1.330	[1.310, 1.350]

eTable 37. Adjusted Risk Ratios for the PASC Diagnosis Subgroup

		Post acute		Chronic	
Category	Symptom	Est	95% CI	Est	95% CI
Signs or symptoms	Abdominal pain	2.824	[2.172, 3.671]	2.157	[1.643, 2.832]
	Bloating	NA[a]	NA	NA	NA
	Constipation	1.573	[1.124, 2.202]	1.09	[0.752, 1.579]
	Diarrhea	1.683	[1.118, 2.533]	NA	NA
	Nausea	NA	NA	NA	NA
	Vomiting	0.93	[0.593, 1.458]	NA	NA
Disorders	GERD	NA	NA	NA	NA
	IBS	NA	NA	NA	NA
	Functional dyspepsia	NA	NA	NA	NA
Composite	Any signs or symptoms	1.753	[1.459, 2.106]	1.468	[1.208, 1.784]
	Any disorders	NA	NA	2.176	[1.387, 3.413]
	Any visits related to GI	1.735	[1.453, 2.070]	1.501	[1.245, 1.809]

[a]: NA means the adjusted risk ratio is not obtained from the model due to the limited sample size for patients with PASC (n=1,388) and rare incidence of GI symptoms and conditions.

eTable 38. Relative Proportion of GI Tract Signs, Symptoms and Disorders to Overall GI Outcomes by Documented SARS-CoV-2 Infection Status[a].

Category	Outcome	Post-acute phase No. (%)[b]		Chronic phase No. (%)	
		COVID-19 positive	COVID-19 negative	COVID-19 positive	COVID-19 negative
Total incident occurrence[c]		45272	99134	71439	147693
Signs and symptoms	Abdominal Pain	9775 (21.59)	22492 (22.69)	17568 (24.59)	37151 (25.15)
	Bloating	1129 (2.49)	2595 (2.62)	1777 (2.49)	3900 (2.64)
	Constipation	10858 (23.98)	25523 (25.75)	17581 (24.61)	40167 (27.20)
	Diarrhea	8946 (19.76)	17453 (17.61)	13011 (18.21)	23821 (16.13)
	Nausea	3274 (7.23)	6398 (6.45)	5438 (7.61)	9802 (6.64)
	Vomiting	11290 (24.94)	24673 (24.89)	16064 (22.49)	32852 (22.24)
Total incident occurrence[c]		5160	13052	7598	16783
Disorders	GERD	4416 (85.58)	10952 (83.91)	6132 (80.71)	13336 (79.46)
	IBS	419 (8.12)	1311 (10.04)	918 (12.08)	2210 (13.17)
	Functional dyspepsia	325 (6.30)	789 (6.05)	548 (7.21)	1237 (7.37)

Abbreviation: GERD, gastroesophageal reflux disease; IBS, irritable bowel syndrome; GI, gastrointestinal.
[a]reported raw numbers and incidence for COVID-19 positive cohort (with documented COVID-19 infection) and COVID-19 negative cohort (without documented COVID-19 infection). Post-acute phase is from 28 days to 179 days after the cohort entry date or the chronic phase is from 180 days to 729 days after the cohort entry date.
[b]Relative proportion is defined as the incident occurrence of specified GI symptoms or disorders during the post-acute or chronic phase, divided by the total incident occurrence of specified GI symptoms or disorders.
[c]Total incident occurrence is defined as the total incident occurrence of specified GI symptoms or disorders during the post-acute or chronic phase

© 2025 Zhang D et al. *JAMA Network Open*.

eReferences.

1. Schuemie MJ, Hripcsak G, Ryan PB, Madigan D, Suchard MA. Empirical confidence interval calibration for population-level effect estimation studies in observational healthcare data. *Proc Natl Acad Sci U S A*. 2018;115(11):2571-2577. doi:10.1073/PNAS.1708282114
2. Schuemie MJ, Ryan PB, Dumouchel W, Suchard MA, Madigan D. Interpreting observational studies: Why empirical calibration is needed to correct p-values. *Stat Med*. 2014;33(2):209-218. doi:10.1002/SIM.5925

© 2025 Zhang D et al. *JAMA Network Open*.
