# Supplementary material for: Pediatric Gastrointestinal Tract Outcomes During the Postacute Phase of COVID-19
Source: JAMA Netw Open. 2025 Feb 7;8(2):e2458366. doi: 10.1001/jamanetworkopen.2024.58366 (PMC11806396; doi:10.1001/jamanetworkopen.2024.58366)
Supplement: Supplement 2. — Nonauthor Collaborators. RECOVER Initiative Members [file jamanetwopen-e2458366-s002.pdf]

Supplemental Online Content: Nonauthor Collaborators
*First name, last name, and suffix (if applicable) are required and will appear in PubMed.

***Group Name(s): RECOVER Initiative Members**

*First Name and Middle Initial(s)	*Last Name	*Suffix (eg, Jr, III)	Academic Degrees	Institution	Location (city, state/province, country)	Role or Contribution, eg, chair, principal investigator	Group (if more than 1 Group listed in the byline) and/or Subgroup (eg, Steering Committee)
Iván	Diaz		PhD	NYU Langone Health	New York, NY, USA	Study Group Lead	
Rachel	Kenney		PhD	NYU Langone Health	New York, NY, USA	Study Group Lead	
Jasmin	Divers		PhD	NYU Langone Health	New York, NY, USA	Study Group Lead (former)	
Lorna	Thorpe		PhD, MPH	NYU Langone Health	New York, NY, USA	Study Group Lead (former)	
Olalekan	Bello		PhD	NYU Langone Health	New York, NY, USA	Query Support	
Yu	Chen		PhD, MPH	NYU Langone Health	New York, NY, USA	Manuscript Support	
Michelle	Lamendola-Essel		DHSC, MS.	NYU Langone Health	New York, NY, USA	Program Director, Observational Studies Operations	
Hannah	Mandel		MPH	NYU Langone Health	New York, NY, USA	Sr. Research Scientist	
Jennifer	Truong		MSc	NYU Langone Health	New York, NY, USA	Sr. Research Project Manager	
Shannon W.	Wuller			NYU Langone Health	New York, NY, USA	Research Project Manager	
Sara J.	Deakyne Davies		MPH	Children's Hospital Colorado	Aurora, CO, USA	Informatics Lead	
Suchitra	Rao		MD	Children's Hospital Colorado	Aurora, CO, USA	Principal Investigator	
L. Charles	Bailey		MD, PhD	Children's Hospital of Philadelphia	Philadelphia, PA	Principal Investigator	
Christopher B.	Forrest		MD, PhD	Children's Hospital of Philadelphia	Philadelphia, PA	Principal Investigator	
Dongkyu	Kim		PhD	Children's National Medical Center	Washington, DC, USA	Informatics Lead	
Nathan M	Pajor		MD	Cincinnati Children's Hospital Medical	Cincinnati, OH, USA	Principal Investigator	
Soumitra	Sengupt		PHD	Columbia University	New York, NY, USA	Principal Investigator	
W. Schuyler	Jones		MD	Duke University Health System	Durham, NC, USA	Principal Investigator	
Kelly J	Kelleher		MD	Nationwide Children's Hospital	Columbus, OH, USA	Principal Investigator	
Yungui	Huang		PhD, MBA	Nationwide Children's Hospital	Columbus, OH, USA	Informatics Lead	
H Timothy	Bunnell		PhD	Nemours Children's Health	Wilmington, DE, USA	Principal Investigator & Informatics Lead	
Maurice	Duque		MS	Nicklaus Children's Hospital	Miami, FL, USA	Informatics Lead	
Nathalia	Ladino		MS	NYU Langone Health	New York, NY, USA	Informatics Lead	
Daniel	Fort		PhD, MPH	Oschner Health	New Orleans, LA, USA	Principal Investigator	
Cynthia H.	Chuang		MD, MSc	Pennsylvania State College of Medicine	Hershey, PA, USA	Principal Investigator	
Daksha	Ranade		MPH, MBA	Seattle Children's Hospital	Seattle, WA, USA	Informatics Lead	
Alan	Schroeder		MD	Stanford University	Palo Alto, CA, USA	Principal Investigator	
Susan	Kim		MD, MMSc	University of California San Fransisco	San Fransisco, CA, USA	Principal Investigator	

Supplemental Online Content: Nonauthor Collaborators
*First name, last name, and suffix (if applicable) are required and will appear in PubMed.

*First Name and Middle Initial(s)	*Last Name	*Suffix (eg, Jr, III)	Academic Degrees	Institution	Location (city, state/province, country)	Role or Contribution, eg, chair, principal investigator	Group (if more than 1 Group listed in the byline) and/or Subgroup (eg, Steering Committee)
Jiang	Bian		PhD, MS	University of Florida	Gainesville, FL, USA	Informatics Lead	
Elizabeth A.	Chrischilles		PHD	University of Iowa	Iowa City, IA, USA	Principal Investigator	
David A.	Williams		PHD	University of Michigan	Ann Arbor, MI, USA	Principal Investigator	
Abu Saleh Mohammad	Mosa		PhD, MS, FAMIA	University of Missouri School of Medicine	Columbia, MO, USA	Principal Investigator	
Carol	Reynolds Geary		PHD, MBA, RN	University of Nebraska Medical Center	Omaha, NE, USA	Principal Investigator	
Michael J.	Becich		MD, PHD	University of Pittsburgh	Pittsburgh, PA, USA	Principal Investigator	
Jonathan	Arnold		MD	University of Pittsburgh	Pittsburgh, PA, USA	Principal Investigator	
Yalini	Senathirajah		PHD	University of Pittsburgh	Pittsburgh, PA, USA	Principal Investigator	
Brian	Ostasiewski			Wake Forest University Health Sciences	Winston Salem, NC, USA	Informatics Lead	
Stephen M.	Downs		MD, MS	Wake Forest University Health Sciences	Winston Salem, NC, USA	Principal Investigator	
Rainu	Kaushal		MD, MPH	Weill Cornell Medicine	New York, NY, USA	Principal Investigator	
Thomas R	Campion	JR	PhD, MS	Weill Cornell Medicine	New York, NY, USA	Informatics Lead	
Selvin	Soby		Pharm.D	Albert Einstein College of Medicine	Bronx, NY	Informatics Lead	
Ravi J	Jhaveri		MD	[illegible] Robert H. Lurie Children's Hospital of Ch[illegible]	Chicago, IL, USA	Principal Investigator	
Jyothi Priya Alekapatti	Nandagopal			Cincinnati Children’s Hospital Medical Cent[illegible]	Cincinnati, OH, USA	Informatics Lead	
Curtis	Kieler			Duke University Health System	Durham, NC, USA	Informatics Lead	
Bradley W	Taylor		FAMIA	The Medical College of Wisconsin	Milwaukee, WI	Principal Investigator	
Alexander	Stoddard		MS	The Medical College of Wisconsin	Milwaukee, WI	Informatics Lead	
Reza	Shaker		MD	The Medical College of Wisconsin	Milwaukee, WI	Principal Investigator	
Saul	Blecker		MD	NYU Langone Health	New York, NY, USA	Principal Investigator	
Marion R.	Sills		MD, MPH	OCHIN, Inc.	Portland, OR, USA	Principal Investigator	
Dimitri A .	Christakis		MD, MPH	Seattle Children's Research Institute	Seattle, WA, USA	Principal Investigator	
Keith E.	Morse		MD, MBA	Stanford University School of Medicine	Palo Alto, CA, USA	Informatics Lead	
Mark J.	Pletcher		MD, MPH	University of California San Fransisco	San Fransisco, CA, USA	Informatics Lead	
Mei	Liu		PHD, MS	University of Florida	Gainesville, FL, USA	Principal Investigator	
Jim	Svoboda		MS	University of Nebraska Medical Center	Omaha, NE, USA	Informatics Lead	
Nickie	Cappella			University of Pittsburgh	Pittsburgh, PA, USA	Informatics Lead	

Supplemental Online Content: Nonauthor Collaborators
*First name, last name, and suffix (if applicable) are required and will appear in PubMed.

*First Name and Middle Initial(s)	*Last Name	*Suffix (eg, Jr, III)	Academic Degrees	Institution	Location (city, state/province, country)	Role or Contribution, eg, chair, principal investigator	Group (if more than 1 Group listed in the byline) and/or Subgroup (eg, Steering Committee)
Wei-Qi	Wei		MD, PhD, FAMIA	Vanderbilt University Medical Center	Nashville, TN, USA	Informatics Lead	
