# Supplementary material for: Pediatric Gastrointestinal Tract Outcomes During the Postacute Phase of COVID-19
Source: JAMA Netw Open. 2025 Feb 7;8(2):e2458366. doi: 10.1001/jamanetworkopen.2024.58366 (PMC11806396; doi:10.1001/jamanetworkopen.2024.58366)
Supplement: Supplement 3. — Data Sharing Statement [file jamanetwopen-e2458366-s003.pdf]

Data Sharing Statement

Zhang. Pediatric Gastrointestinal Tract Outcomes During the Postacute Phase of COVID-19. *JAMA Netw Open*. Published February 06, 2025. doi:10.1001/jamanetworkopen.2024.58366

Data
Data available: Yes
Data types: Deidentified participant data
How to access data: For more information on RECOVER, visit https://recovercovid.org/.
When available: With publication

Supporting Documents
Document types: None

Additional Information
Who can access the data: researchers whose proposed use of the data has been approved
Types of analyses: for a specified purpose
Mechanisms of data availability: after approval of a proposal
